# Compartment-specific metabolome labeling enables the identification of subcellular fluxes that may serve as promising metabolic engineering targets in CHO cells

**DOI:** 10.1007/s00449-021-02628-1

**Published:** 2021-09-30

**Authors:** Andy Wiranata Wijaya, Andreas Ulmer, Lara Hundsdorfer, Natascha Verhagen, Attila Teleki, Ralf Takors

**Affiliations:** Institute of Biochemical Engineering, University of Stuttgart, Allmandring 31, 70569 Stuttgart, Germany

**Keywords:** Compartment-specific, Metabolomics, ^13^C Metabolic flux analysis, Chinese hamster ovary cells, Eukaryotes, Multi-compartments

## Abstract

^13^C labeling data are used to calculate quantitative intracellular flux patterns reflecting in vivo conditions. Given that approaches for compartment-specific metabolomics exist, the benefits they offer compared to conventional non-compartmented ^13^C flux studies remain to be determined. Using compartment-specific labeling information of IgG1-producing Chinese hamster ovary cells, this study investigated differences of flux patterns exploiting and ignoring metabolic labeling data of cytosol and mitochondria. Although cellular analysis provided good estimates for the majority of intracellular fluxes, half of the mitochondrial transporters, and NADH and ATP balances, severe differences were found for some reactions. Accurate flux estimations of almost all iso-enzymes heavily depended on the sub-cellular labeling information. Furthermore, key discrepancies were found for the mitochondrial carriers *v*_AGC1_ (Aspartate/Glutamate antiporter), *v*_DIC_ (Malate/H^+^ symporter), and *v*_OGC_ (α-ketoglutarate/malate antiporter). Special emphasis is given to the flux of cytosolic malic enzyme (*v*_ME_): it could not be estimated without the compartment-specific malate labeling information. Interesting enough, cytosolic malic enzyme is an important metabolic engineering target for improving cell-specific IgG1 productivity. Hence, compartment-specific ^13^C labeling analysis serves as prerequisite for related metabolic engineering studies.

**Supplementary Information:**

The online version contains supplementary material available at 10.1007/s00449-021-02628-1.

## Introduction

^13^C metabolic flux analysis (^13^C MFA) is a key tool for quantitative analysis in systems metabolic engineering. First, applications dealt with prokaryotic cells [1] but the technique was also applied for eukaryotes, such as yeast [2, 3], fungi [4], mammalian [5–8], and plant [9] cells. Among others, prokaryotes and eukaryotes differ in cellular compartmentation, which is particularly important when using ^13^C MFA. In eukaryotes, compartmentation is essential since each cellular compartment fulfils different functions [10]. Even multi-compartment isozymes exist that serve different purposes. For example, Chinese hamster ovary (CHO) cells comprise cytosolic and mitochondrial malic enzymes (MEs) with different NAD^+^ and NADP^+^ regeneration capacities, thereby fulfilling diverse catabolic and anabolic needs [8].

Metabolic compartmentation must be considered when performing ^13^C MFA [10]. There are two levels of complexity; on the one hand, subcellular metabolic models should be used to enable proper in silico predictions. On the other hand, in vivo compartment-specific metabolome data should be available to allow data-driven studies. Nicolae et al. and Pfizenmaier & Takors provided evidence for the importance of subcellular stoichiometric models for estimating fluxes in CHO cells [11, 12]. Regarding the latter, Matuszczyk et al. [13] applied compartment-specific metabolomics in CHO outlining that cytosolic ATP pools are considerably larger than their mitochondrial counterparts. Later, Junghans et al. [8] continued investigating mitochondrial and cytosolic metabolic patterns under different cultivation conditions. They found that pool sizes differed between cytosol and mitochondria for all conditions.

Given that subcellular metabolomics are very laborious [8, 13] the question arises what differences may occur if ^13^C flux analysis is based on whole-cell metabolomics instead of compartment-specific measurements. In other words, whether the additional lab-efforts justify the information gain of subcellular studies.

Alternative approaches such as superimposing the patterns of two independent ^13^C experiments using labeled glucose and labeled glutamine also aim to decipher subcellular flux distributions [6]. However, they rely on glutamine synthase deficient cells whereas the suggested subcellular metabolomics approach may be universally applicable.

Given that labeling dynamics in metabolite pools expressed by the ^13^C labeling turn-over (τ_13C_) are a key information for quantifying fluxes, influencing factors may be considered. Two factors, pool size of metabolite *i* and net labeling flux *j* through this pool exist [14]. Either factor may change when a system’s analysis shifts from simplifying single to realistic multi-compartment analysis. Differences in τ_13C_ may occur originating from individual pool sizes and fluxes inside the compartments. In theory, the same metabolite in different compartment might present a different labeling dynamic providing that the metabolite turn-over time is different. Thus, resulting on a different labeling dynamics (τ_13C_).

Exploiting the unique subcellular labeling dataset of Junghans et al. [8] this study investigated whether subcellular labeling information is crucial to obtain the correct compartment-specific flux patterns. Flux distributions considering and ignoring subcellular metabolite labeling were performed using CHO as the showcase. This study investigated whether significant differences exist using whole-cell and compartment-specific metabolic information.

## Materials and methods

This study was based on published metabolome and ^13^C isotopologue data [8]. In particular, the ^13^C dataset covering the first 24 h was used to focus on the exponential growth phase.

### Cell culture and experimental set-up

The CHO DP-12 cell line (ATCC^®^ CRL-1445TM) was cultivated in a suspension with TC-42 medium (Xell AG, Bielefeld, Germany) supplemented with 42 mM d-glucose, 6 mM ʟ-glutamine, and 200 mM methotrexate. Precultures were cultivated in pre-sterilized disposable shake flasks (Corning Inc., NY, USA) with culture volume ranging from 125 mL to 1 L at an initial viable cell density (VCD) of 0.4 × 10^6^ cells/mL in a humidified shaking incubator (Infors HT Minitron, Infors GmbH, Einsbach, Germany) at 37 °C, 150 rpm, and 5% saturated CO_2_.

Bioreactor cultivations were performed in a two-fold parallel CellFerm Pro bioreactor system (DASGIP, Eppendorf, Germany) equipped with pitched blade impellers and a process control system. Bioreactor cultivations were started with a VCD of about 0.4 × 10^6^ cells/mL, temperature was set to 37 °C and agitation to 150 rpm. In addition, the dissolved oxygen content was controlled using an amperometric electrode (Mettler-Toledo Inc., Columbus, OH, USA) at 40%. The pH was measured with a conventional pH probe (Mettler-Toledo Inc., Columbus, OH, USA) and maintained at 7.1 using 1 M Na_2_CO_3_ or CO_2_ gassing. Carbon labeling experiments were performed in the same setup using [U-^13^C_6_]-d-glucose as a carbon tracer with an average isotopic ratio of 25% [U-^12^C_6_]- and 75% [U-^13^C_6_]-d-glucose. Experiments were performed as biological duplicates. In addition to carbon labeling experiments, bioreactor cultivations with [U-^12^C_6_]-d-glucose were performed using the same conditions for metabolome profiling.

### Extracellular and intracellular analytics

VCD was monitored with a 12 h interval with Cedex XS, an offline cell counting system (Innovatis AG, Bielefeld, Germany). Extracellular d-glucose and ʟ-lactate were monitored offline with LaboTRACE, an amperometric biosensor system (Trace Analytics GmbH, Braunschweig, Germany). Extracellular antibody (IgG1) concentrations were measured using ELISA as reported previously [15]. Extracellular amino acid concentrations were quantified with reversed-phase chromatography (Agilent 1200 Series, Agilent Technologies, Waldbronn, Germany) [8].

Sampling for metabolomics was performed using differential fast filtration [8, 13]. Then, processed samples were analyzed regarding metabolome quantification using an Agilent 1200 HPLC system coupled with an Agilent 6410B (Agilent Technologies, Waldbronn, Germany) triple quadrupole mass spectrometer equipped with an electrospray ion source. Analytical sample preparation and methodology were conducted as reported previously [8, 16].

### ^13^C metabolic flux analysis

**Fig. 1:**
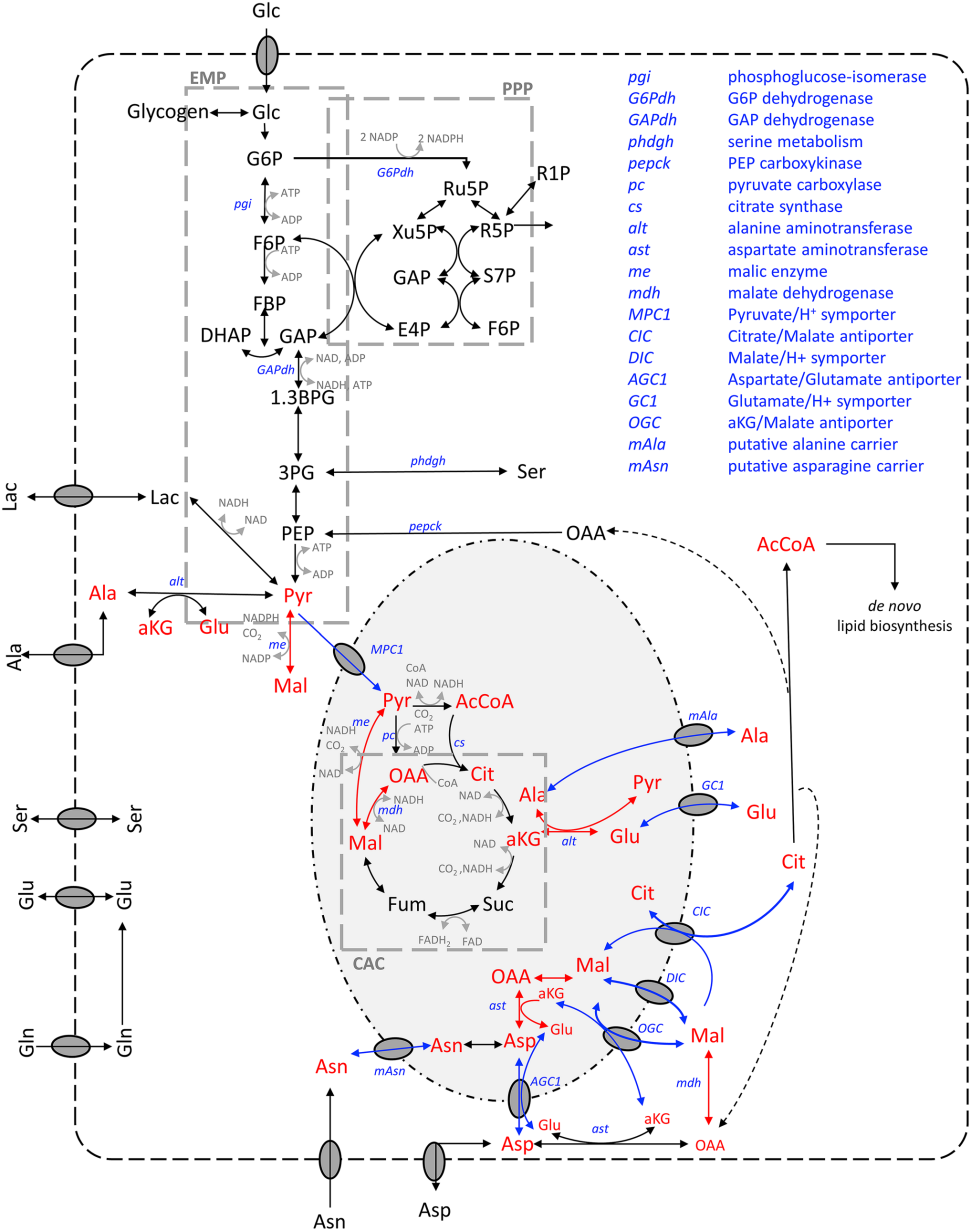
Metabolic model of CHO cells used in this study (modified figure from Junghans et al. [8]). Arrow coloring indicates the localization of biochemical reactions as follows: black encodes single compartment; red encodes multi-compartments; and blue encodes inter-compartment transporters. In addition, multi-compartment metabolites are indicated in red (color figure online)

Isotopic non-stationary ^13^C MFA was performed in MATLAB 2018a (The MathWorks, Inc., MA, USA). Before performing ^13^C MFA, measured ^13^C labeling distributions were corrected for natural stable isotope abundances [17]. Parameter optimization was conducted using MATLAB least square optimization *fmincon* function in combination with *GlobalSearch* and *MultiStart* algorithm in a multi-core computing machine [18]. The first derivative of each isotopomer balance was solved using MATLAB Ordinary Differential Equations *ode15s* solver. The study used the metabolic and carbon-atom transition model in the previous study [8]. Details of the model are indicated in Table S1 (Supplementary Material S1) and are displayed in Fig. 1.

#### Metabolite balancing

The two-compartment CHO-cell model comprises the stoichiometric matrix *S* (Supplementary Material S1, Table S1) consisting of *m* metabolites and *n* reactions (*m* × *n*). The following cell-specific rates [pmol cell^−1^ h^−1^] were defined: *q* for cellular uptake and secretion rates, *k* as inter-compartment transport, and *v* as compartment-specific reaction. The balance of metabolite *i* participating in reaction *j* localized externally, in cytosol, or in mitochondria was described by Eqs. 1 and 2:

1 $$\frac{{d_{{C_{{i,{\text{ex}}}} }} }}{dt} = Q_{{i,{\text{feed}}}} + q_{i} c_{X} ,$$

2 $$\frac{{d_{{c_{{i,{\text{in}}}} }} }}{dt} = \left( { - q_{i} - k_{i} + \mathop \sum \limits_{j = 1}^{n} v_{j} } \right) \cdot c_{x} = 0,$$

where *c*_*i*_ denotes the concentration of metabolite *i* [mol L^−1^], *c*_*x*_ denotes VCD [cell L^−1^], *t* denotes time [h], and *Q*_*i,*feed_ denotes the feed-rate of metabolite *i* [pmol L^−1^ h^−1^].

The process model describing the batch cultivation is given in Eq. 1 and allows the estimation of *q* for metabolite *i* by time-series analysis of extracellular concentrations *c*_*i*_. Therefore, the metabolic steady-state was defined as mirrored in the constraint $\frac{{d}_{c,\mathrm{intracellular}}}{dt}=0$, which is a prerequisite for ^13^C flux analysis. Both stationary and non-stationary labeling patterns were analyzed, originating from the metabolic steady-state condition.

#### Metabolic flux analysis

MFA was performed using the metabolic network *S* considering the following constraints: (i) pool sizes of cytosolic and mitochondrial metabolites were in a steady-state and (ii) the entire system was (over)-determined because of the ample ^13^C labeling information. Fluxes were estimated according to:

3 $$v = \left( {\begin{array}{*{20}c} S \\ M \\ \end{array} } \right)^{ - 1} \left( {\begin{array}{*{20}c} 0 \\ {\left[ {\begin{array}{*{20}c} {q_{{{\text{meas}}}} } & p \\ \end{array} } \right]} \\ \end{array} } \right),$$

where *M* is the measurement matrix containing the stoichiometric coefficients of *q*_meas_ (measured rates [pmol cell^−1^ h^−1^]) and *p* contains the estimated fluxes using mass-isotopomer data [pmol cell^−1^ h^−1^]).

#### Isotopomer balancing and bidirectional reactions

Isotopomer balancing was applied to mathematically describe the incorporation of ^13^C tracers into intracellular metabolite carbon skeletons [19, 20]. Isotopomer balances for intracellular metabolites are according to Eq. 4:

4 $$\begin{gathered} \frac{{d\left( {{\mathbf{C}}_{{\text{i}}} {\mathbf{I}}_{{\mathbf{i}}} } \right)}}{dt} = \mathop \sum \limits_{j = 1}^{N} \left[ {\alpha \left( {\begin{array}{*{20}c} 0 \\ \otimes \\ {k = 1} \\ \end{array} \left( {\mathop \sum \limits_{m = 1}^{n} {\mathbf{IMM}}_{k \to m} } \right){\mathbf{I}}_{k} } \right)r_{j} + \left( {1 - \alpha } \right)\left( {v_{i} r_{j} {\mathbf{I}}_{i} } \right)} \right] \hfill \\ {\text{with}} \hfill \\ \alpha = \left\{ {\begin{array}{*{20}c} {1, {\text{if}} v_{ij} > 0} \\ {0, {\text{else}} } \\ \end{array} } \right., \hfill \\ \end{gathered}$$

where the isotopomer transition from reactant *k* to product *m* is described by **IMM**_*k*→*m*_.

Furthermore, Eq. 5 was used to describe labeling dilution by extracellular pools (ʟ-lactate, ʟ-glutamate, ʟ-aspartate, and ʟ-alanine):

5 $$\begin{gathered} \frac{{d\left( {{\mathbf{I}}_{{i,{\text{ex}}}} } \right)}}{dt} = \frac{1}{{c_{{i,{\text{ex}}}} }}\left[ {\overline{{c_{X} }} \left( {\overset{\lower0.5em\hbox{$\smash{\scriptscriptstyle\rightharpoonup}$}}{{q_{{i,{\text{ex}}}} }} \cdot {\mathbf{I}}_{{i,{\text{in}}}} - \overset{\lower0.5em\hbox{$\smash{\scriptscriptstyle\leftharpoonup}$}}{{q_{{i,{\text{ex}}}} }} \cdot {\mathbf{I}}_{{i,{\text{ex}}}} } \right) - \frac{{dc_{{i,{\text{ex}}}} }}{dt}{\mathbf{I}}_{{i,{\text{ex}}}} } \right] \hfill \\ {\text{with}} \hfill \\ \overset{\lower0.5em\hbox{$\smash{\scriptscriptstyle\rightharpoonup}$}}{{q_{{i,{\text{ex}}}} }} = \beta_{i} \cdot q_{{i,{\text{ex}}}}^{{{\text{net}}}} \hfill \\ \overset{\lower0.5em\hbox{$\smash{\scriptscriptstyle\leftharpoonup}$}}{{q_{{i,{\text{ex}}}} }} = \overset{\lower0.5em\hbox{$\smash{\scriptscriptstyle\rightharpoonup}$}}{{q_{{i,{\text{ex}}}} }} - q_{{i,{\text{ex}}}}^{{{\text{net}}}} . \hfill \\ \end{gathered}$$

Exchange fluxes were defined for each reversible biochemical reaction [21, 22] according to Eq. 6:

6 $$\begin{gathered} \overset{\lower0.5em\hbox{$\smash{\scriptscriptstyle\rightharpoonup}$}}{{v_{j} }} = \beta_{j} \cdot v_{j}^{{{\text{net}}}} \hfill \\ \overset{\lower0.5em\hbox{$\smash{\scriptscriptstyle\leftharpoonup}$}}{{v_{j} }} = \overset{\lower0.5em\hbox{$\smash{\scriptscriptstyle\rightharpoonup}$}}{{v_{j} }} - v_{j}^{{{\text{net}}}} . \hfill \\ \end{gathered}$$

#### Parameter estimation and uncertainty

Parameter (flux) estimation was achieved by fitting the simulated mass isotopomer distribution (MID) to the measured in vivo MID as presented in Eq. 7:

7 $$\min f\left( \Phi \right) = \sum \left( {\frac{{{\text{MID}}_{i}^{{{\text{sim}}}} - {\text{MID}}_{i}^{\exp } }}{{\sigma_{i} }}} \right)^{2} .$$

Cytosolic and mitochondrial MIDs were defined for subcellular studies. Non-compartmented analysis considered that no subcellular measurements were available. Instead, only entire cell labeling patterns should exist. Consequently, compartment-specific information was merged again, applying Eq. 8:

8 $${\text{MID}}_{i}^{{{\text{comb}}}} = {\text{MID}}_{i}^{{{\text{cyt}}}} \cdot f + {\text{MID}}_{i}^{{{\text{mit}}}} \cdot \left( {1 - f} \right),$$

where *f* denotes the molar fraction of metabolite *i* in the cytosol. During simulations, *f* was treated as an optimization parameter for those metabolites presented in both compartments; pyruvate, citrate, α-ketoglutarate, malate, alanine, aspartate, asparagine, and glutamine. Accordingly, *f* serves as an alternate indicator for the importance of considering compartments properly. Furthermore, flux estimation was achieved by fitting the measured non-compartment metabolome data with calculated MID using Eq. 9:

9 $$\min f\left( \Phi \right) = \sum \left( {\frac{{{\text{MID}}_{i}^{{{\text{comb}}}} - {\text{MID}}_{i}^{\exp } }}{{\sigma_{i} }}} \right)^{2} .$$

A χ^2^ statistical test was used to assess goodness of fit as described in Eq. 10:

10 $$\begin{gathered} \chi^{2} = \sum \frac{{\left( {x^{{{\text{sim}}}} - x^{\exp } } \right)^{2} }}{{\sigma^{2} }} \hfill \\ dof = \left( {n - p} \right) \hfill \\ \chi^{2} \le \chi_{{\left( {1 - \alpha } \right), dof.}}^{2} \hfill \\ \end{gathered}$$

Parameter uncertainty is essential to evaluate the flux differences including versus excluding compartment-specific data. Conventional parameter uncertainty estimates make use of the local calculation of the Jacobian matrix as a linearized proxy for variance. However, this approach only shows poor performance if a complex and non-linear set of equations should be analyzed, as it is the case in this ^13^C MFA study. Thus, confidence intervals of each parameter (fluxes) were estimated using the Chi-squared (χ^2^) statistics, which works best for non-linear equations as demonstrated by Antoniewicz et al. [23]. The method relies on the assumption that the minimized variance-weighted sum of squared residuals is χ^2^ distributed. Thus, the residual difference evaluating the global optimum and fixing one parameter is χ^2^ distributed with one degree of freedom.

### Statistical analysis

The significant differences between the two analyses were assessed using Welch’s *t*-test for unequal variances [24].

## Results

Prior to the ^13^C MFA studies, a metabolic network model was formulated (Supplementary Material S1). First the structural identifiability and calculability of the network was assessed applying well established methodologies (Supplementary Material S4). Next, the identifiability of distinct fluxes was checked by simulating intracellular ^13^C labeling patterns assuming pool sizes measured by Junghans et al. [8]. Results presented in the Supplementary Material S4 indicate the good identifiability of intracellular fluxes which motivated us to continue the study by analyzing real labeling patterns and flux distributions.

In the study by Junghans et al. [8] CHO-DP12 cells were cultivated in a bioreactor to investigate three distinct growth scenarios; (I) exponential growth with no (carbon and nitrogen) limitation; (II) moderate growth with ʟ-glutamine depletion and ʟ-asparagine saturation; and (III) stationary phase with severe nitrogen limitation. However, the current study regarding the impact of subcellular ^13^C data only covers the exponential growth phase during the first 24 h. This period is typically investigated in vitro because labeling and cultivation conditions can be controlled easily, giving accurate results regarding flux distributions and cell-specific productivities [5, 7]. Furthermore, additional cultivation study data investigating the same cell line and process conditions was used for broadening the data set of subcellular versus cellular ^13^C metabolomics for flux analysis (see Supplementary Material S6). The summary of all estimated intracellular fluxes is provided in Supplementary Material S2.

### Cell growth and carbon labeling studies

During the exponential growth phase, cells grew with 0.025 ± 0.001 h^−1^. Carbon and nitrogen sources were constantly consumed, and metabolic byproducts were steadily released with constant specific rates (Supplementary Material S1, Table S2). d-Glucose was consumed as a major carbon source while ʟ-glutamine and ʟ-asparagine served as primary nitrogen sources. In addition, the Warburg effect [25] was observed, showing a glucose-to-lactate ratio of 0.93 mol_d-glucose_/mol_l-lactate_. ^13^C carbon labeling was introduced by the addition of 75% [U-^13^C_6_]-d-glucose after 2.5 days, revealing no phenotypic changes, i.e., no alterations of cellular metabolism.

### ^13^C metabolic flux analysis using compartment-specific metabolome data

^13^C MFA was performed using compartment-specific metabolome data reflecting subcellular pools of cytosol and mitochondria together with isotopomer profiles of the said compartments. Flux estimations were performed at least 100 times with randomized input values and rational boundary values for each parameter (Supplementary Material S2). Finally, the chi-square tests achieved 228.87, which served the statistical constraint of 232.92 on a 95% significance level.

#### Glycolysis and PPP

High glycolytic (0.112 ± 0.017 pmol cell^−1^ h^−1^ of hexokinase) and extremely low PPP fluxes (0.008 ± 0.001 pmol cell^−1^ h^−1^ of G6P dehydrogenase) were found. The latter accounted for 6.68% of the d-glucose consumed. These observations are in agreement with the findings of Ahn & Antoniewicz [5], who performed ^13^C MFA in adherent CHO-K1 cells. In addition, approximately 15% (0.016 ± 0.002 pmol cell^−1^ h^−1^) of intracellular G6P was continuously in exchange with endogenous glycogen.

#### In vivo mitochondrial shuttle

Glycolytic carbon fueled into mitochondria via two transport mechanisms; 77% entered via the mitochondrial pyruvate carrier (MPC1/2) and 23% via a putative l-alanine transporter. MPC1/2 showed the highest mitochondrial transport activities while other transporters exchanged compounds for different purposes; (i) mitochondrial citrate carrier (citrate/malate antiporter; 0.049 ± 0.002 pmol cell^−1^ h^−1^) served as a citrate exporter to provide cytosolic acetyl-CoA for the de novo lipid biosynthesis pathway; (ii) the malate-aspartate shuttle comprising 2-oxoglutarate carrier (α-ketoglutarate/mal antiporter) and aspartate-glutamate carrier (aspartate/glutamate antiporter), which is often described as an indirect NADH shuttle because imported malate is oxidized to oxaloacetate, releasing NADH, fulfilled a different function; malate was net exported from mitochondria to fuel cytosolic ME.

#### Cytosolic malic enzyme and NADPH production

NADPH is a key electron donator for anabolic pathways and is essential for monoclonal antibody biosynthesis. Ahn & Antoniewicz, Templeton et al. [5, 7] suggested MEs as key NADPH producers in CHO cells. This hypothesis was further confirmed via compartment-specific flux analysis by Junghans et al. [8]. Cytosolic ME (ME_cyt_) was identified as the primary provider serving NADPH needs. Compartment-specific ^13^C MFA estimated that about 86% of the NADPH requirement was fulfilled by ME_cyt_ (0.09 ± 0.01 pmol cell^−1^ h^−1^).

### ^13^C Metabolic flux analysis using non-compartmented metabolome data

An additional ^13^C MFA was performed to investigate the importance of distinct sub-cellular information to elucidate proper in vivo subcellular flux patterns. Analyzing the merged data (Eq. 6) via ^13^C MFA yielded a Chi-squared value of 140.12 on the 95% confidence level, which was accepted as a good fit (with 154.30 as the χ^2^ statistical threshold on 95% confidence interval).

**Fig. 2:**
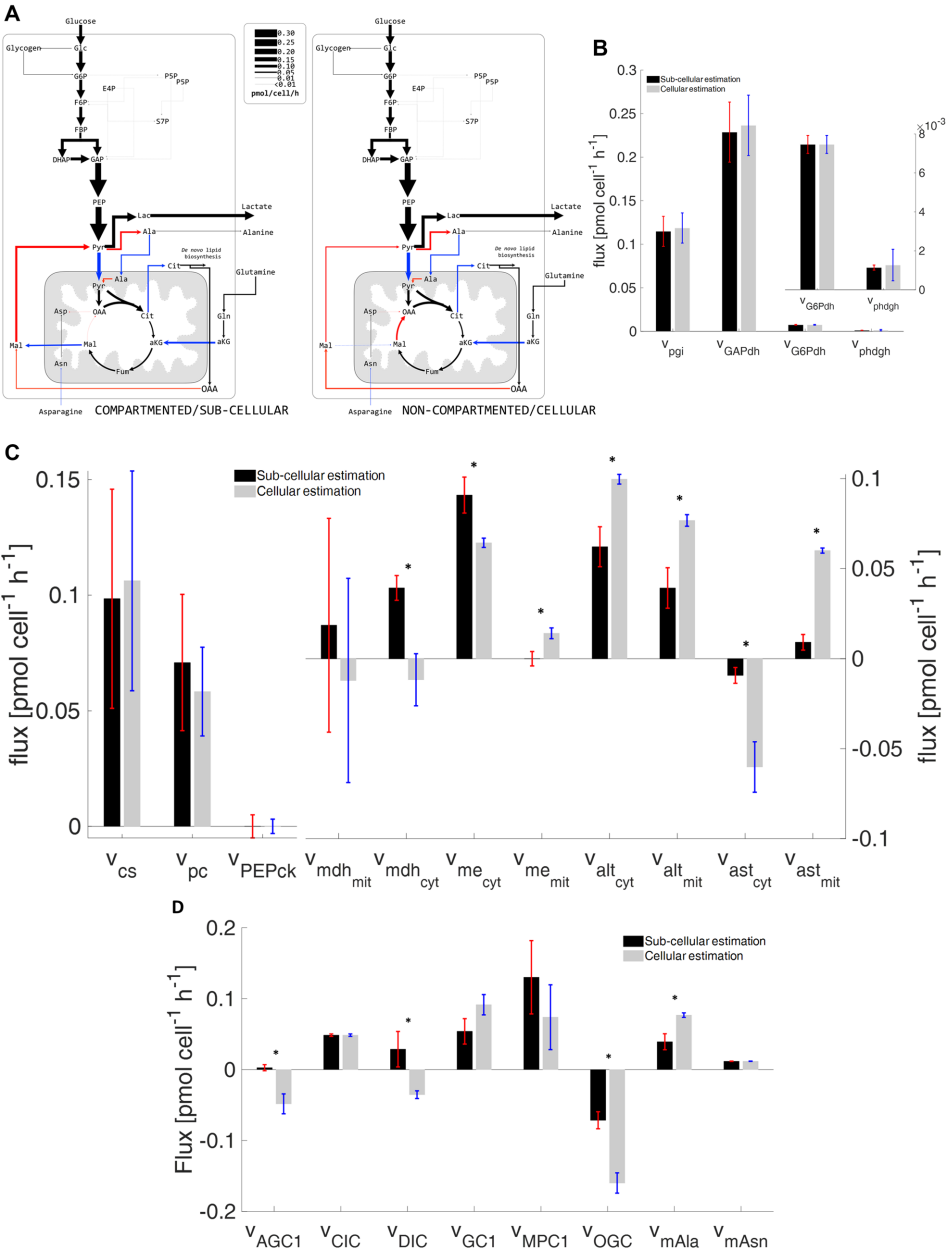
**A** Intracellular flux distribution estimated using compartment-specific (left) and non-compartmented data (right); **B** fluxes of biochemical reactions involving single-compartment metabolites; **C** fluxes of biochemical reactions involving multi-compartment metabolites; and **D** mitochondrial carrier fluxes estimated with compartment-specific and non-compartmented data (* indicates significance *p* < 0.05)

This study was performed using the same model consisting of 42 intracellular biochemical reactions. Figure 2A provides the comparison of intracellular flux distributions estimated with (left) and without (right) sub-cellular information (Fig. 2A). The related single-compartment key fluxes and iso-enzymatic rates are depicted as bar plots in Fig. 2B, C. Notably, the term ‘iso enzymes’ encodes fluxes connecting the same substrates and products but localized in different compartments.

#### Biochemical reactions localized in a single compartment

Figure 2B, C left shows fluxes of biochemical reactions that exist in one compartment (cytosol or mitochondria) only. Most of them revealed similar results irrespective of whether compartment-specific information was used (black) or not (gray). Figure 2B demonstrates the case the metabolome pools and the respective fluxes were the same for both studies, yielding a similar τ_13C_. This is also true for citrate synthase *v*_CS_, although identifiability was poor. Similar results were observed for cytosolic-based reactions: pyruvate carboxylase (*v*_pc_) and PEP carboxykinase (*v*_pepck_) (Fig. 2C). These single-compartment reactions possessed the particularity of utilizing the same metabolites but in different compartments (Fig. 1). In this particular case, no statistically sound difference between *v*_pc_ and *v*_pepck_ was found, most likely because compartment-specific OAA values lacked.

**Fig. 3:**
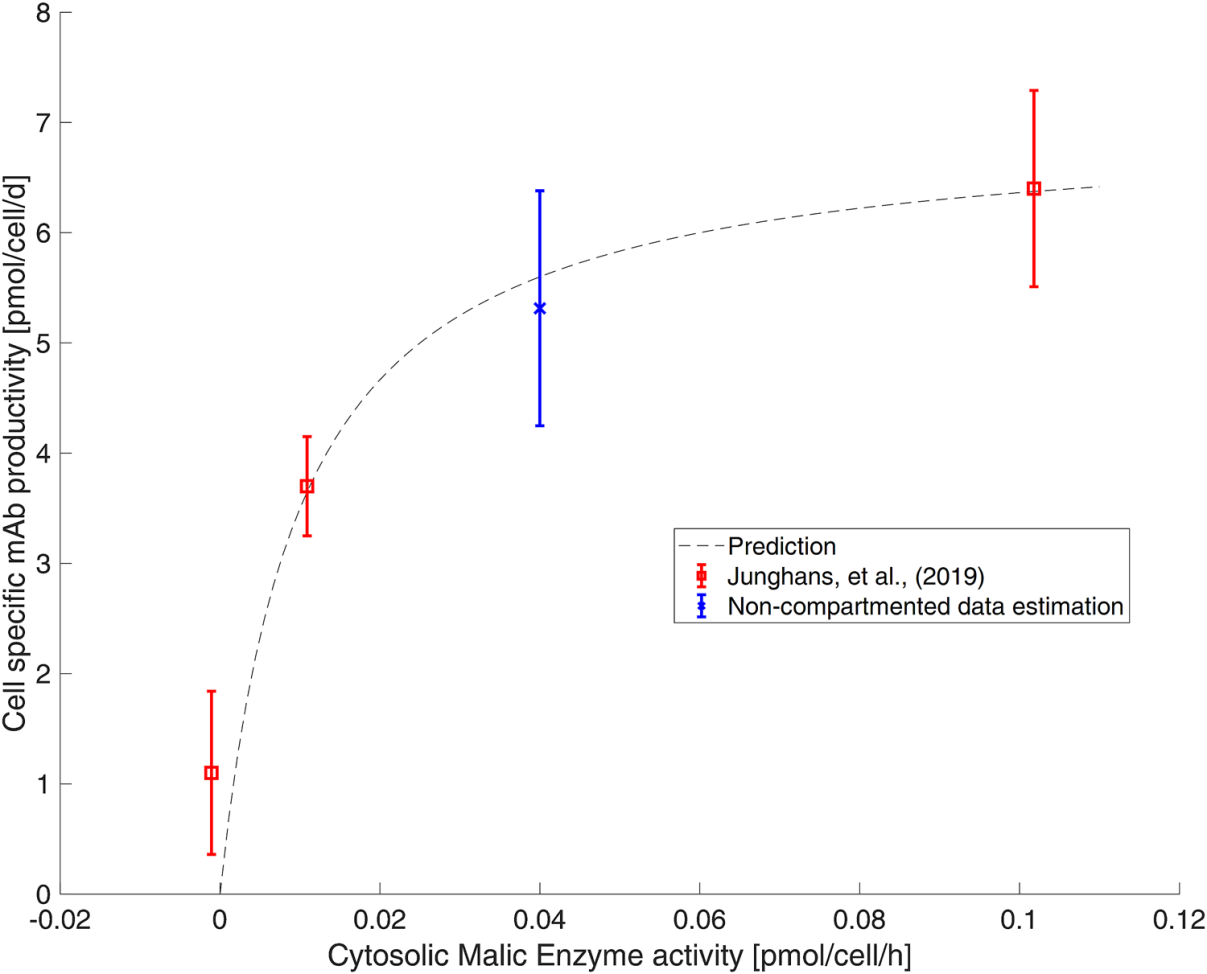
Cell-specific production of monoclonal antibodies in CHO cells (modified from Junghans et al. [8])

#### Iso-enzymatic reactions localized in different compartments

Special emphasis is laid on the so-called iso-enzymatic reactions of Fig. 2C right that catalyze similar conversions in different compartments. The fluxes of malate dehydrogenase (*v*_mdh_), ME (*v*_me_), aspartate amino-transferases (*v*_ast_), and alanine amino-transferases (*v*_alt_) are localized in cytosol and mitochondria, respectively. Of the eight iso-enzymes analyzed, seven conversion rates were significantly different. The only exception is the mitochondrial malate dehydrogenase (*v*_mdh,mit_) which revealed statistical similarity although fluxes even reversed. On contrary, the cytosolic malate dehydrogenase (*v*_mdh,cyt_) also disclosed flux reversion but with a sound statistical identifiability.

Non-compartmented data were not able to properly reflect real fluxes of the amino-transferases (*v*_ast_), namely alanine amino-transferases (*v*_alt_) and aspartate amino transferases (*v*_ast_). The analysis of whole-cell data resulted in flux overestimation compared to compartment-specific analysis. Notably, the substrate aspartate occurred in cytosol and mitochondria and is a key player of the aspartate-malate shuttle. Moreover, alanine was involved in the co-transport of glycolytic carbon into mitochondria with the MPC1/2. In this case, proper localization and labeling information of the compound is key to estimate fluxes correctly.

In addition, severe bias was observed for fluxes of both malic enzymes (*v*_me_) as displayed in Fig. 2C right. By trend, ^13^C flux estimations using non-compartmented data identified significantly lower (about 30%) cytosolic *v*_me,cyt_ than the non-compartmented data. Regarding mitochondria, the opposite was found. The finding for *v*_me_ using non-compartmented data is consistent with the observations of Ahn & Antoniewicz, Templeton et al. [5, 7] who also performed ^13^C MFA with cellular data. Importantly, cytosolic ME activity via *v*_me,cyt_ was identified as a key supplier for NADPH needed for IgG production in CHO cells (Junghans et al. [8]).

#### Mitochondrial metabolite carriers

Comparing shuttle activities using sub-cellular and cellular labeling information reveals significant differences for half of the inter-compartment transporters, namely the aspartate/glutamate antiporter (*v*_AGC1_), malate carrier (*v*_DIC_), α-ketoglutarate/malate antiporter (*v*_OGC_), and the putative alanine carrier (*v*_mAla_) (Fig. 2D). Similar to the identification of aspartate amino-transferases, the proper identification of *v*_AGC1_ depends on the labeling turnover τ_13C_ of Asp in both compartments. Missing compartment-specific measurements lead to the different shuttle fluxes, which are also reflected in the biased flux *v*_ast_. The same scenario also holds true for the putative alanine carrier (*v*_mAla_) and the corresponding reactions (alanine amino-transferases; *v*_alt_). Shuttle estimations regarding *v*_DIC_ and *v*_OGC_ using non-compartment-specific data contradict flux calculations using compartment-specific information estimation. The sub-cellular labeling information of malate is essential to get accurate flux estimates. Interestingly, the flux estimation of putative asparagine carrier (*v*_mAsn_) was not biased by the use of whole-cell labeling data only. This may reflect that *v*_mAsn_ heavily depends on the measured ʟ-asparagine uptake rate (*q*_Asn_) irrespective of the existence of additional subcellular information.

#### Estimated cytosol–mitochondrial fraction (*f* factor)

Using Eq. 8, *f* factors were estimated for each metabolite and compared with the measurements of Junghans et al. [8] (Table 1). As indicated, all estimated cytosolic fractions (*f*) were poorly identified with pyruvate showing the smallest difference of 8.59% only. On average, 59.71% difference was found compared to the real labeling fraction. Notably, the best estimates of pyruvate and asparagine also enabled improved flux values for the corresponding biochemical reactions, e.g. *v*_MPC1/2_, *v*_pdh_ for pyruvate, and *v*_asns_, *v*_mAsn_ for asparagine.

**Table 1:** Complete list of estimated and measured cytosolic fractions of subcellular metabolites used for 13C MFA

Metabolites	Cytosolic fraction (*f*)		
	Estimated	Measurement (Junghans et al. [8])	% difference (measurement as the reference value)
Mal	0.100	0.829	87.9
Pyr	0.910	0.838	8.59
aKG	0.100	0.714	85.99
Cit	0.995	0.489	103.48
Glu	0.373	0.827	54.90
Ala	0.100	0.840	88.1
Asn	0.717	0.805	10.48
Asp	0.500	0.809	38.20

#### Cellular NADH and NADPH production

Table 2 shows a comparison of NADH and NADPH production via compartment-specific analysis and neglection of sub-cellular data.

**Table 2:** Comparison of NADH, ATP, and NADPH net production rates in compartment-specific analysis and whole-cell analysis (values presented in pmol cell^−1^ h^−1^)

	NADH	ATP	NADPH
Compartment-specific	0.55692	0.22752	0.10577
Non-compartmented	0.60815	0.25914	0.07924

Neglecting sub-cellular data, NADPH production is underestimated by approximately 25%. This reflects the 30% underestimation of cytosolic *v*_ME_ when cellular and not subcellular data are used. In the case of NADH and ATP, the utilization of different datasets disclosed only minor differences. NADH and ATP fluxes were overestimated by 9% and 14% for non-compartmented data, respectively.

#### Challenging the key statements by an additional data set

To investigate whether or not the observed flux characteristics may be specific for the data sets used, additional data of cultivations with the same cell line, cultivation conditions, and analytical tools was used. Figure S6-1:S6-3 (Supplementary Material S6) outlines that very similar key messages are obtained analyzing the new data set: Glycolytic fluxes are fairly similar irrespective whether subcellular or cellular ^13^C metabolomics is used. On contrary, fluxes for cytosolic malate dehydrogenase and malic enzyme differ statistically significant depending on the granularity of metabolic labeling resolution. The same holds true for shuttle activities such as DIC, GC1, and OGC which is in agreement with the results derived from the other data sets.

## Discussion

This study challenges the information gain when performing ^13^C MFA with compartment-specific metabolome data compared to exploiting cellular labeling information not distinguishing between cytosol and mitochondria.

Figure 2 outlines the complexity of the interactions. A group of fluxes (*v*_pgi_, *v*_GAPdh_* v*_G6Pdh_, and *v*_phdgh_) located in a single compartment (here: cytosol) disclose equal values irrespective of the analytical approach selected. Interestingly, this also holds true for *v*_cs_, located in mitochondria, primarily due to poor flux identifiability. Furthermore, *v*_pepck_ and *v*_pc_ revealed such high flux variances that no distinction could be found whether cellular or subcellular ^13^C data were used. Apparently, both reactions depend on cytosolic (OAA_cyt_) and mitochondrial oxaloacetate (OAA_mit_). They act at the interphase of the two compartments and rely on proper sub-cellular measurement information (τ_13C_) for correct identification. Distinct OAA measurements were not available in the current study due to challenging analytical access to the compound. Accordingly, flux estimations might be biased by the quality of OAA pool estimations.

In addition, some other fluxes should be interpreted with great care, too. This holds particularly true for mitochondrial malate dehydrogenase (*v*_mdh,mit_) and the pyruvate carrier *v*_MPC1_. Both disclose large error bars rendering a discrimination between cellular versus subcellular approaches hardly possible (Fig. 2C, D). Flux imprecisions reflect the lack of reliable CO_2_ evolution rates ($q_{{{\text{CO}}_{2} }}$) and CO_2_ labeling profiles.

The whole-cell (cellular) flux estimation failed to estimate the mitochondrial and cytosolic fluxes of the amino-transferases *v*_alt_ and *v*_ast_. This may reflect that those fluxes heavily depend on the compartment-specific labeling information of alanine and aspartate. Not providing this information by using whole-cell labeling data leads to the large discrepancies given in Fig. 2C.

Almost all mitochondrial carrier fluxes were poorly estimated when using non-compartmented data. Inaccurate estimations of *v*_AGC1_ and *v*_mAla_ are also reflected by the results of *v*_ast_ and *v*_alt_. In addition, the poor estimation of the malate carriers *v*_DIC_ and *v*_OGC_ depended on *v*_me_. In general, fluxes of transporters and bioreactions heavily relied on the labeling dynamics measured in the related metabolites. Regarding *v*_MPC1_, the reduced shuttle activity based on non-compartmented data reflects the missing malate exported into cytosol (Fig. 2D).

To check whether the additional use of labeled glutamine [6] might have achieved similar subcellular flux resolutions as the compartment-specific analysis, simulations were performed using [U-^13^C_5_]-ʟ-glutamine (Supplementary Material S3). Interestingly, without information about compartment-specific metabolomics, cytosolic ^13^C signals obtained from simulations are pretty similar to those of the whole-cell. This is mainly due to the relatively low information gain with respect to the key mitochondrial metabolites malate and aspartate. Compartment-specific labeling information and turnover of the latter are decisive to resolve activities of mitochondrial transporters.

In general, most of the flux estimations using either non-compartmented or compartmented data led to similar values. Even global cell qualifications, such as rates of total ATP formation and NADH production, were similar. However, two main findings should be considered:

1. Often, cellular analysis achieved similar flux estimations as subcellular studies by fitting measured cytosolic labeling fractions for the sake of estimating pool sizes properly (Table 1). In other words, flux optimization algorithms adapted cytosolic and mitochondrial pool sizes to complement missing labeling information. However, the simulated pool size readouts were strongly misleading.
2. Among the fluxes with the largest discrepancies is the cytosolic ME *v*_me_. Remarkably, this flux was found to be a promising metabolic engineering target to maximize the formation of heterologous proteins by improved NADPH supply [8]. Accordingly, exact estimation is a prerequisite for proper strain engineering. Figure 3 illustrates that even the result of non-compartment data analysis still fits to the subcellular kinetics published in Junghans et al. [8]. Whether or not experimentalists may have identified this enzyme as a metabolic engineering target remains open and is a matter of qualitative discussion rather than quantitative target identification [8].

To date, the compartment-specific analytical approach has shown its suitability for multiple metabolomic studies investigating CHO cells under in vivo-like conditions [8, 15, 24–30]. The latter is enabled by fast and standardized metabolism inactivation. Furthermore, data quality essentially relies on the quantitative access to internal standards, such as G6P/F6P (in cytosolic space) and *cis*-aconitate (in mitochondrion) to correct for mitochondrial leakage. In general, fast metabolic inactivation, standardized sample processing and use of internal standards are prerequisites for any compartment-specific metabolomics approach that might be used in future applications.

## Conclusions

Investigating the need for using subcellular ^13^C labeling data, the study revealed that non-compartmented data enabled to identify most fluxes involving single compartment metabolites. Besides, half of the mitochondrial shuttle fluxes and global properties, such as ATP and NADH formation, were fairly well estimated without requiring further subcellular labeling information. However, there is a number of sensitive fluxes that could only be identified properly if compartment-specific pool information was used. Among those were mitochondrial shuttles that rely on alanine, aspartate and malate. Furthermore, key metabolic engineering targets, such as the cytosolic ME flux for NADPH formation, were severely underestimated using (total) cellular data. This may disguise their role as promising metabolic engineering target if non-compartmented pool analysis is performed, only. The finding underlines the necessity to apply subcellular data for flux estimation, not only to quantify cytosolic/mitochondrial shuttle activities but also to identify metabolic engineering targets and obtain valid values for real pool sizes.

## Supplementary Information

Below is the link to the electronic supplementary material.

Supplementary file1 (PDF 137 kb)

Supplementary file2 (PDF 158 kb)

Supplementary file3 (PDF 169 kb)

Supplementary file4 (PDF 1935 kb)

Supplementary file5 (PDF 73 kb)

Supplementary file6 (DOCX 178 kb)

## Abbreviations

^13^C MFA: ^13^C metabolic flux analysis
CHO: Chinese hamster ovary
VCD: Viable cell density
PPP: Pentose phosphate pathway
CAC: Citric acid cycle
MID: Mass isotopomer distribution
MPC1/2: Mitochondrial pyruvate carrier
ME: Malic enzyme

## Symbols

*c*_*i*_: pmol L^−^^1^ Concentration of metabolite *i*
*c*_*x*_: cell L^−^^1^ Viable cell density
*dof*: [-] Degree of freedom
*E*: [-] Expected MID measurement data
*f*_*i*_: [-] Cytosolic fraction of metabolite *i*
**I**: [-] Isotopomer distribution vector
**IMM**: [-] Isotopomer mapping matrices
MID: [-] Mass isotopomer distribution
*n*: [-] Number of measurement data
*O*: [-] Observed MID simulation
**p**: pmol cell^−^^1^ h^−^^1^ Vector containing estimated fluxes using mass-isotopomers data
*p*: [-] Number of fitted parameter
*Q*_*i*_: pmol L^−^^1^ h^−^^1^ Feed-rate of metabolite *i*
*q*_*i*_: pmol cell^−^^1^ h^−^^1^ Cell-specific rate of exo-metabolite *i*
**q**_**m**_: pmol cell^−^^1^ h^−^^1^ Vector containing measured extracellular rates
*S*: [-] Stoichiometric matrix of biochemical network
*M*: [-] Measurement information matrix
*v*_*j*_: pmol cell^−^^1^ h^−^^1^ Intracellular flux of biochemical reaction *j*
**v**: pmol cell^−^^1^ h^−^^1^ Vector containing intracellular metabolic fluxes

## Greek symbols

*α*: [-] Statistical confidence interval
*β*: [-] Reversibility constant
*σ*: [-] Measurement standard deviation of MID
Θ: [-] Parameter

## Indices

ex: [-] Compartment indication
feed: [-] Feed
in: [-] Compartment indication
*i*: [-] Compound/metabolite *i*
*j*: [-] Biochemical reactions *j*
meas: [-] Indication for measurement vector
net: [-] Indication for net fluxes
X: [-] Cells/biomass
