# Supplementary material for: Compartment-specific metabolome labeling enables the identification of subcellular fluxes that may serve as promising metabolic engineering targets in CHO cells
Source: Bioprocess Biosyst Eng. 2021 Sep 30;44(12):2567–78. doi: 10.1007/s00449-021-02628-1 (PMC8536584; doi:10.1007/s00449-021-02628-1)
Supplement: Supplementary file 1 — Supplementary file1 (PDF 137 kb) [file 449_2021_2628_MOESM1_ESM.pdf]

Table S1: Metabolic and carbon atom transition model used in this study

ID	Enzyme name	Reaction and carbon atom transition
tGln	Glutamine importer	Gln_ex[(1,2,3,4,5)] => Gln[(1,2,3,4,5)]
gs4	Glutaminase	Gln[(1,2,3,4,5)] => Glu[(1,2,3,4,5)]
tGlu	Glutamate transporter	Glu[(1,2,3,4,5)] = Glu_ex[(1,2,3,4,5)]
Gdh	Glutamate dehydrogenase	aKG_m[(1,2,3,4,5)] = Glu_m[(1,2,3,4,5)]
tGlc	Glucose importer	Glc_ex[(1,2,3,4,5,6)] => G6P[(1,2,3,4,5,6)]
fGlyco	Carbon storage (glycogen) degradation	Glyco_ex[(1,2,3,4,5,6)] => G6P[(1,2,3,4,5,6)]
Pgi	Phospho-glucose-isomerase	G6P[(1,2,3,4,5,6)] = F6P[(1,2,3,4,5,6)]
Pfk	Phosphofructokinase + fructose-1,6-bisphosphatase	F6P[(1,2,3,4,5,6)] = FBP[(1,2,3,4,5,6)]
fbpa	Fructose-bisphosphate aldolase	FBP[(1,2,3,4,5,6)] = DHAP[(3,2,1)] + GAP[(4,5,6)]
Tpi	Triophosphate isomerase	DHAP[(1,2,3)] = GAP[(1,2,3)]
gapdh	GAP dehydrogenase + biphosphoglycerate mutase	GAP[(1,2,3)] = 3PG[(1,2,3)]
Eno	Phosphoglycerate hydratase	3PG[(1,2,3)] = PEP[(1,2,3)]
pkm	Pyruvate kinase	PEP[(1,2,3)] => Pyr[(1,2,3)]
Ldh	Lactate dehydrogenase	Pyr[(1,2,3)] = Lac[(1,2,3)]
tLac	Lactate transporter	Lac[(1,2,3)] => Lac_ex[(1,2,3)]
G6Pdh	Glucose-6-phosphate dehydrogenase	G6P[(1,2,3,4,5,6)] => Ru5P [(2,3,4,5,6)] + CO2[(1)]
Rpi	Ribulose-5-phosphate isomerase	Ru5P[(1,2,3,4,5)] = R5P[(1,2,3,4,5)]
tkt1	Transketolase	R5P[(1,2,3,4,5)(6,7,8,9,10)] = S7P[(6,7,1,2,3,4,5)] + GAP[(8,9,10)]
Tald	Transaldolase	E4P[(1,2,3,4)] + R5P[(5,6,7,8,9)] = F6P[(5,6,1,2,3,4)] + GAP[(7,8,9)]
tkt2	Transketolase	S7P[(1,2,3,4,5,6,7)] + GAP[(8,9,10)] = E4P[(4,5,6,7)] + F6P[(1,2,3,8,9,10)]
tCO2	CO_2 evolution	CO2[(1)] => CO2_ex[(1)]
MPC1	Pyruvate/H^+ symporter	Pyr[(1,2,3)] => Pyr_m[(1,2,3)]
CIC	Citrate/Malate antiporter	Cit_m[(1,2,3,4,5,6)] + Mal[(7,8,9,10)] => Cit[(1,2,3,4,5,6)] + Mal_m[(7,8,9,10)]
DIC	PO_3^{4-}/Malate antiporter	Mal_m[(1,2,3,4)] = Mal[(1,2,3,4)]
GC1	Glutamate/H^+ symporter	Glu_m[(1,2,3,4,5)] = Glu[(1,2,3,4,5)]
OGC	Malate/aKG antiporter	Mal[(1,2,3,4)] + aKG_m[(5,6,7,8,9)] = Mal_m[(1,2,3,4)] + aKG[(5,6,7,8,9)]
AGC1	Aspartate/glutamate antiporter	Glu[(1,2,3,4,5)] + Asp_m[(6,7,8,9)] => Glu_m[(1,2,3,4,5)] + Asp[(6,7,8,9)]
mAla	Putative alanine transporter	Ala[(1,2,3)] = Ala_m[(1,2,3)]

mAsn	Putative asparagine transporter	Asn[(1,2,3,4)] = Asn_m[(1,2,3,4)]
Pdh	Pyruvate dehydrogenase	Pyr_m[(1,2,3)] => AcCoA_m[(2,3)] + CO2[(1)]
Cs	Citrate synthase	OAA_m[(1,2,3,4)] + AcCoA_m[(5,6)] => Cit_m[(4,3,2,6,5,1)]
Idh	Iso-citrate dehydrogenase	Cit_m[(1,2,3,4,5,6)] = aKG_m[(1,2,3,4,5)] + CO2[(6)]
Adh	aKG dehydrogenase + succinyl-CoA ligase + succinate dehydrogenase + fumarase	aKG_m[(1,2,3,4,5)(6,7,8,9,10)] = Mal_m[(2,3,4,5)(10,9,8,7)] + CO2[(1)(6)]
Mdh	Malate dehydrogenase	Mal_m[(1,2,3,4)(5,6,7,8)] = OAA_m[(1,2,3,4)(8,7,6,5)]
Pepck	Phosphoenolpyruvate carboxykinase	OAA[(1,2,3,4)] = PEP[(1,2,3)] + CO2[(4)]
me_c	Cytosolic malic enzyme	Mal[(1,2,3,4)] = Pyr[(1,2,3)] + CO2[(4)]
me_m	Mitochondrial malic enzyme	Mal_m[(1,2,3,4)] = Pyr_m[(1,2,3)] + CO2[(4)]
Pc	Pyruvate carboxylase	Pyr_m[(1,2,3)] + CO2[(4)] = OAA_m[(1,2,3,4)]
mdh_c	Cytosolic malate dehydrogenase	OAA[(1,2,3,4)(5,6,7,8)] = Mal[(1,2,3,4)(8,7,6,5)]
tSer	Serine importer	Ser_ex[(1,2,3)] => Ser[(1,2,3)]
phdgh	Phosphoglycerate dehydrogenase + phosphoserine phosphatase	3PG[(1,2,3)] => Ser[(1,2,3)]
sds	Serine dehydratase	Ser[(1,2,3)] => Pyr[(1,2,3)]
tAla	Alanine transporter	Ala[(1,2,3)] = Ala_ex[(1,2,3)]
alt_c	Cytosolic alanine aminotransferase	Pyr[(1,2,3)] + Glu[(4,5,6,7,8)] = Ala[(1,2,3)] + aKG[(4,5,6,7,8)]
alt_m	Mitochondrial alanine aminotransferase	Ala_m[(1,2,3)] + aKG_m[(4,5,6,7,8)] = Pyr_m[(1,2,3)] + Glu_m[(4,5,6,7,8)]
tAsp	Aspartate transporter	Asp_ex[(1,2,3,4)] = Asp[(1,2,3,4)]
tAsn	Asparagine importer	Asn_ex[(1,2,3,4)] => Asn[(1,2,3,4)]
ast_c	Cytosolic aspartate aminotransferase	Asp[(1,2,3,4)] + aKG[(5,6,7,8,9)] = OAA[(1,2,3,4)] + Glu[(5,6,7,8,9)]
ast_m	Mitochondrial aspartate aminotransferase	Asp_m[(1,2,3,4)] + aKG_m[(5,6,7,8,9)] = OAA_m[(1,2,3,4)] + Glu_m[(5,6,7,8,9)]
asns	Asparaginase	Asn_m[(1,2,3,4)] = Asp_m[(1,2,3,4)]
muG6P	Biomass formation from G6P	G6P[(1,2,3,4,5,6)] => G6P_X_ex[(1,2,3,4,5,6)]
muGAP	Biomass formation from GAP	GAP[(1,2,3)] => GAP_X_ex[(1,2,3)]
muRu5P	Biomass formation from Ru5P	R5P[(1,2,3,4,5)] => R5P_X_ex[(1,2,3,4,5)]
acl	Acetyl-CoA lyase	Cit[(1,2,3,4,5,6)] => OAA[(6,3,2,1)] + AcCoA[(5,4)]
muAcCoA	Biomass formation from AcCoA	AcCoA[(1,2)] => AcCoA_X_ex[(1,2)]
muSer	Biomass formation from	Ser[(1,2,3)] => Ser_X_ex[(1,2,3)]

	serine	
muAla	Biomass formation from alanine	Ala[(1,2,3)] => Ala_X_ex[(1,2,3)]
muAsp	Biomass formation from aspartate	Asp[(1,2,3,4)] => Asp_X_ex[(1,2,3,4)]
muAsn	Biomass formation from asparagine	Asn[(1,2,3,4)] => Asn_X_ex[(1,2,3,4)]
muGln	Biomass formation from glutamine	Gln[(1,2,3,4,5)] => Gln_X_ex[(1,2,3,4,5)]
muGlu	Biomass formation from glutamate	Glu[(1,2,3,4,5)] => Glu_X_ex[(1,2,3,4,5)]

Table S2: Specific uptake and release rates of extracellular metabolites and its Lower Boundary (LB) and Upper Boundary (UB)

**Metabolites**	**Uptake/Secretion rate (pmol cell^{-1} h^{-1})[1]**	**[LB UB][2]**
D-glucose	-0.112	[-0.129 -0.095]
L-lactate	0.134	[0.106 0.163]
L-glutamine	-0.057	[-0.067 -0.047]
L-glutamate	0.023	[0.014 0.032]
L-asparagine	-0.017	[-0.017 -0.017]
L-aspartate	0.003	[0.002 0.004]
L-alanine	0.011	[0.009 0.013]
L-serine	-0.016	[-0.011 -0.021]
IgG_1	0.443[3]	[0.354 0.532][3]

[1] value < 0 means uptake; value > 0 means secretion
[2]according to 95% confidence interval (linear regression)
[3]unit is in pg cell^{-1} h^{-1}
