# Supplementary material for: Compartment-specific metabolome labeling enables the identification of subcellular fluxes that may serve as promising metabolic engineering targets in CHO cells
Source: Bioprocess Biosyst Eng. 2021 Sep 30;44(12):2567–78. doi: 10.1007/s00449-021-02628-1 (PMC8536584; doi:10.1007/s00449-021-02628-1)
Supplement: Supplementary file 2 — Supplementary file2 (PDF 158 kb) [file 449_2021_2628_MOESM2_ESM.pdf]

METABOLIC FLUX DISTRIBUTION

	Flux ID	Reaction	COMPARTMENT-SPECIFIC				NON-COMPARTMENTED				Welsch Test	
			mean	error prop	UB (95%)	LB (95%)	mean	error prop	UB (95%)	LB (95%)	p	Significance
**Extracellular rates**	tAla	Ala <-> Ala_ex	0.011	0.0017	0.013	0.009	0.011	0.0017	0.013	0.009		
	tAsn	Asn_ex -> Asn	0.017	0.0000	0.017	0.017	0.017	0.0000	0.017	0.017		
	tAsp	Asp <-> Asp_ex	0.003	0.0010	0.004	0.002	0.003	0.0010	0.004	0.002		
	tCO2	CO2 -> CO2_ex	0.246	0.1529	0.399	0.093	0.269	0.1532	0.422	0.116		
	tGlc	Glc -> G6P	0.112	0.0171	0.129	0.095	0.112	0.0171	0.129	0.095		
	tGln	Gln_ex -> Gln	0.057	0.0104	0.067	0.047	0.057	0.0104	0.067	0.047		
	tGlu	Glu <-> Glu_ex	0.023	0.0094	0.032	0.014	0.023	0.0094	0.032	0.014		
	tLac	Lac <-> Lac_ex	0.134	0.0288	0.163	0.106	0.134	0.0288	0.163	0.106		
	tSer	Ser_ex -> Ser	0.016	0.0050	0.021	0.011	0.016	0.0050	0.021	0.011		
**Biomass growth**	acl	Cit --> OAA + AcCoA	0.049	0.0018	0.050	0.047	0.049	0.0018	0.050	0.047		
	muAcCoA	AcCoA -> AcCoA_X	0.049	0.0018	0.050	0.047	0.049	0.0018	0.050	0.047		
	muAla	Ala -> Ala_X	0.012	0.0004	0.012	0.011	0.012	0.0004	0.012	0.011		
	muAsn	Asn -> Asn_X	0.006	0.0002	0.006	0.005	0.006	0.0002	0.006	0.005		
	muAsp	Asp -> Asp_X	0.009	0.0003	0.010	0.009	0.009	0.0003	0.010	0.009		
	muG6P	G6P -> G6P_X	0.006	0.0002	0.006	0.005	0.006	0.0002	0.006	0.005		
	muGAP	GAP -> GAP_X	0.002	0.0001	0.002	0.002	0.002	0.0001	0.002	0.002		
	muGln	Gln -> Gln_X	0.006	0.0002	0.007	0.006	0.006	0.0002	0.007	0.006		
	muGlu	Glu -> Glu_X	0.008	0.0003	0.008	0.007	0.008	0.0003	0.008	0.007		
	muP5P	P5P -> P5P_X	0.007	0.0002	0.007	0.006	0.007	0.0002	0.007	0.006		
	muSer	Ser -> Ser_X	0.009	0.0003	0.009	0.008	0.009	0.0003	0.009	0.008		
**EMP**	fGlyco	Glyco -> G6P	0.016	0.0022	0.020	0.013	0.020	0.0028	0.021	0.017	0.1576	NON-SIG
	pgi	G6P <-> F6P	0.115	0.0173	0.132	0.098	0.119	0.0173	0.136	0.101	0.8018	NON-SIG
	pfk	F6P <-> FbP	0.115	0.0172	0.133	0.098	0.119	0.0173	0.137	0.102	0.8018	NON-SIG
	fbpa	FbP <-> GAP + DHAP	0.115	0.0172	0.133	0.098	0.119	0.0173	0.137	0.102	0.8018	NON-SIG
	tpi	DHAP <-> GAP	0.115	0.0172	0.133	0.098	0.119	0.0173	0.137	0.102	0.8018	NON-SIG
	gapdh	GAP <-> 3PG	0.229	0.0345	0.263	0.194	0.237	0.0347	0.271	0.202	0.8018	NON-SIG
	eno	3PG <-> PEP	0.228	0.0345	0.262	0.193	0.235	0.0347	0.270	0.201	0.8018	NON-SIG
	pkm	PEP -> Pyr	0.228	0.0349	0.262	0.193	0.235	0.0348	0.270	0.200	0.8018	NON-SIG
	ldh	Pyr <-> Lac	0.134	0.0288	0.163	0.106	0.134	0.0288	0.163	0.106	1	NON-SIG
[illegible]	G6Pdh	G6P -> P5P + CO2	0.007	0.0005	0.008	0.007	0.007	0.0005	0.008	0.007	1	NON-SIG

PPP	tkt1	2*P5P <-> S7P + GAP	0.000	0.0002	0.000	0.000	0.000	0.0002	0.000	0.000	1	NON-SIG
	tald	E4P + P5P <-> F6P + GAP	0.000	0.0002	0.000	0.000	0.000	0.0002	0.000	0.000	1	NON-SIG
	tkt2	S7P + GAP <-> E4P + F6P	0.000	0.0002	0.000	0.000	0.000	0.0002	0.000	0.000	1	NON-SIG
Mitochondrial shuttles	AGC1	Glu + Asp_m --> Glu_m + Asp	0.003	0.0043	0.007	-0.002	-0.048	0.0140	-0.034	-0.062	0.0264	SIG
	CIC	Cit_m + Mal --> Cit + Mal_m	0.049	0.0018	0.050	0.047	0.049	0.0018	0.050	0.047	1	NON-SIG
	DIC	Mal_m <-> Mal	0.029	0.0250	0.054	0.004	-0.035	0.0054	-0.030	-0.041	0.0492	SIG
	GC1	Glu_m <-> Glu	0.054	0.0179	0.072	0.036	0.091	0.0143	0.106	0.077	0.0660	NON-SIG
	MPC1	Pyr --> Pyr_m	0.130	0.0517	0.182	0.078	0.074	0.0457	0.119	0.028	0.252	NON-SIG
	OGC	Mal + aKG_m <-> Mal_m + aKG	-0.071	0.0120	-0.059	-0.083	-0.160	0.0143	-0.146	-0.174	0.0038	SIG
	mAla	Ala <-> Ala_m	0.039	0.0113	0.050	0.028	0.077	0.0032	0.080	0.074	0.0309	SIG
	mAsn	Asn <-> Asn_m	0.012	0.0002	0.012	0.012	0.012	0.0002	0.012	0.012	1	NON-SIG
	mCO2	CO2_m <-> CO2	0.148	0.1545	0.302	-0.007	0.197	0.1533	0.351	0.044	0.7187	NON-SIG
Citric acid	pdh	Pyr_m -> AcCoA_m	0.098	0.0474	0.146	0.051	0.106	0.0475	0.154	0.059	0.8269	NON-SIG
	csyn	AcCoA + OAA -> Cit	0.098	0.0474	0.146	0.051	0.106	0.0475	0.154	0.059	0.8269	NON-SIG
	idh	Cit_m <-> aKG_m + CO2_m	0.050	0.0474	0.097	0.003	0.058	0.0475	0.105	0.010	0.827	NON-SIG
	adh	aKG_m <-> Mal_m + CO2_m	0.070	0.0533	0.123	0.017	0.078	0.0533	0.131	0.025	0.8461	NON-SIG
	mdh	Mal_m <-> OAA_m	0.019	0.0594	0.078	-0.041	-0.012	0.0567	0.045	-0.069	0.1518	NON-SIG
	mdhc	OAA <-> Mal	0.039	0.0069	0.046	0.032	-0.012	0.0145	0.003	-0.026	0.0178	SIG
anaplerotic	pc	Pyr_m <-> OAA_m	0.071	0.0295	0.100	0.041	0.058	0.0192	0.078	0.039	0.5807	NON-SIG
	me	Mal <-> Pyr + CO2	0.091	0.0100	0.101	0.069	0.064	0.0026	0.068	0.062	0.04930	SIG
	mem	Mal_m <-> Pyr_m + CO2_m	0.000	0.0040	0.013	-0.004	0.014	0.0030	0.026	0.011	0.0167	SIG
	pepck	OAA <-> PEP + CO2	0.000	0.0050	0.005	-0.001	0.000	0.0031	0.003	-0.001	1.00000	NON-SIG
Amino acids metabolism	gs	Gln -> Glu	0.051	0.0104	0.061	0.040	0.051	0.0104	0.061	0.040	1	NON-SIG
	gdh	aKG_m <-> Glu_m	-0.003	0.0142	0.011	-0.017	-0.003	0.0142	0.011	-0.017	1	NON-SIG
	phdgh	3PG -> Ser	0.001	0.0001	0.001	0.001	0.001	0.0008	0.002	0.000	0.80950	NON-SIG
	sds	Ser -> Pyr	0.008	0.0050	0.013	0.003	0.008	0.0051	0.013	0.003	0.90768	NON-SIG
	asnsm	Asn_m <-> Asp_m	0.012	0.0002	0.012	0.012	0.012	0.0002	0.012	0.012	1	NON-SIG
	ast	Asp + aKG <-> OAA + Glu	-0.009	0.0044	-0.005	-0.014	-0.060	0.0140	-0.046	-0.074	0.0267	SIG
	astm	Asp_m + aKG_m <-> OAA_m + Glu_m	0.009	0.0043	0.013	0.005	0.060	0.0140	0.074	0.046	0.0264	SIG
	alt	Pyr + Glu <-> Ala + aKG	0.062	0.0111	0.073	0.053	0.100	0.0027	0.102	0.097	0.0296	SIG
	altm	Ala_m + aKG_m <-> Pyr_m + Glu_m	0.039	0.0113	0.050	0.028	0.077	0.0032	0.080	0.074	0.0309	SIG

COFACTORS

	Compartment-specific	Non-compartmented
NADPH	0.10577	0.07924
NADH	0.55692	0.60815
ATP	0.22752	0.25914

METABOLOME POOL SIZES

Metabolite	Pool size (fmol/cell)
Glucose 6-phosphate	2.80
Ribulose 5-phosphate	8.10
Fructose 6-phosphate	0.73
Fructose 1,6-bisphosphat	0.59
Dihydroxyacetone phosp	0.51
2/3-phospho glycerate	0.61
Pyruvate_cyt	2.70
Pyruvate_mit	0.64
Citrate_cyt	3.70
Citrate_mit	3.90
aKG_cyt	0.23
aKG_mit	0.08
Malate_cyt	5.10
Malate_mit	0.99
Glutamine	5.10
Glutamate_cyt	51.00
Glutamate_mit	11.00
Serin	32.00
Alanine_cyt	13.10
Alanine_mit	3.00
Aspartate_cyt	23.30
Asparate_mit	5.10
Asparagine_cyt	22.40
Asparagine_mit	6.00
