# Supplementary figures and images for: Compartment-specific metabolome labeling enables the identification of subcellular fluxes that may serve as promising metabolic engineering targets in CHO cells
Source: Bioprocess Biosyst Eng. 2021 Sep 30;44(12):2567–78. doi: 10.1007/s00449-021-02628-1 (PMC8536584; doi:10.1007/s00449-021-02628-1)

Simulation result when using 100% [U-<sup>13</sup>C<sub>5</sub>]-L-glutamine as the carbon tracer.

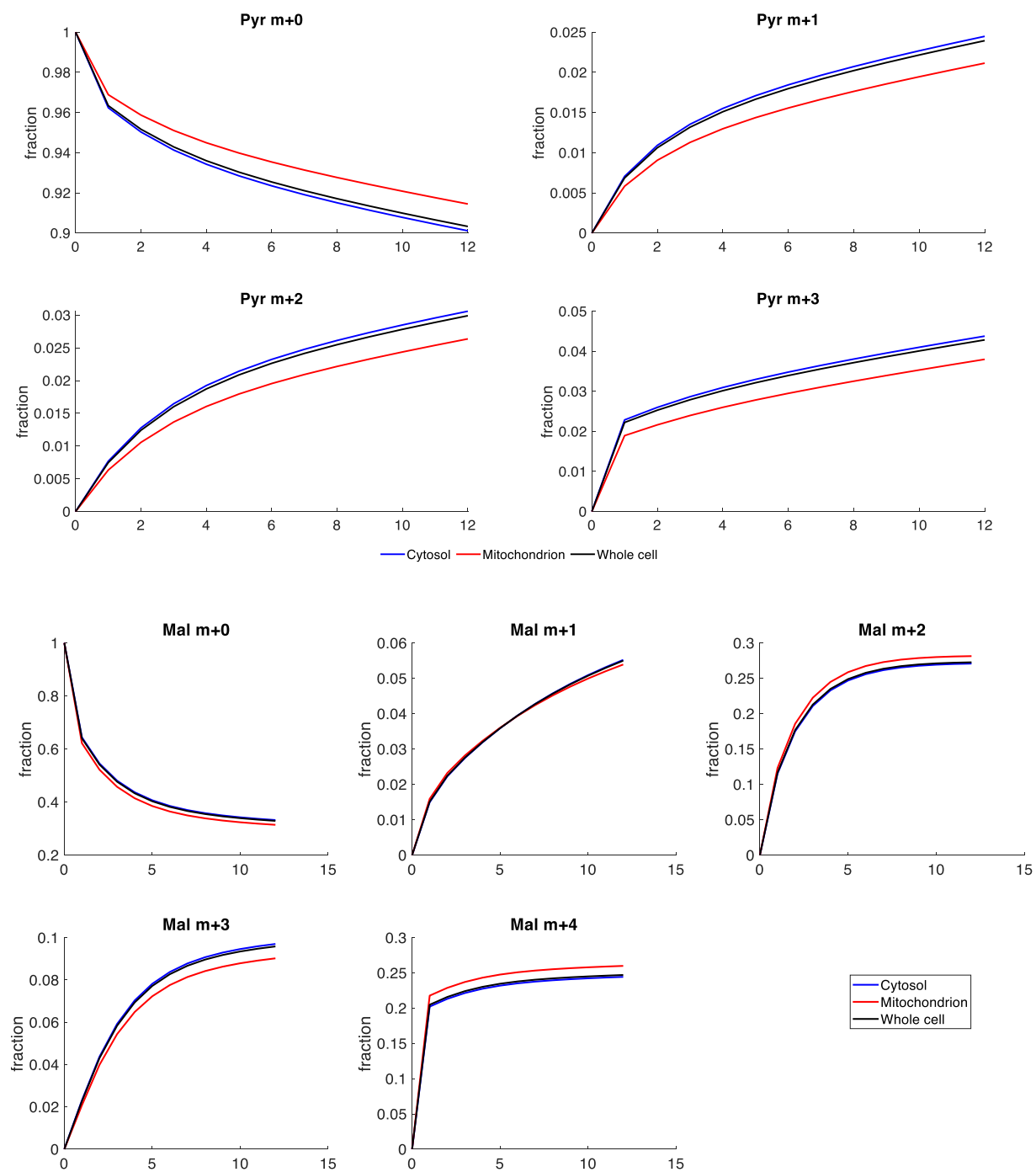

Supplement: Supplementary file 3 — Supplementary file3 (PDF 169 kb) [file 449_2021_2628_MOESM3_ESM.pdf]
