# Supplementary material for: Compartment-specific metabolome labeling enables the identification of subcellular fluxes that may serve as promising metabolic engineering targets in CHO cells
Source: Bioprocess Biosyst Eng. 2021 Sep 30;44(12):2567–78. doi: 10.1007/s00449-021-02628-1 (PMC8536584; doi:10.1007/s00449-021-02628-1)
Supplement: Supplementary file 4 — Supplementary file4 (PDF 1935 kb) [file 449_2021_2628_MOESM4_ESM.pdf]

Supplementary Material S4

I. Network identifiability analysis

The metabolic network (*S*) used in this study consists of 61 reactions *r* and 35 metabolites *m*. The complete metabolic model is presented in Supplementary Material S1.

The network degree of freedom may be calculated as follow:

$$n_{df} = \dim(v) - \mathrm{rank}(S) = 61 - 35 = 26$$

where dim(*v*) is the number of biochemical reactions in network *S*; and rank(S) is the number of independent metabolite balances (without redundancy; e.g. conserved moieties).

The degree of freedom (n_{df}) indicates that at least 26 flux rates are required to quantify the intracellular fluxes in the metabolic network *S*.

Furthermore, calculability analysis (Klamt et al., 2002) was performed to assess how many reactions can be calculated from the input free fluxes. In this case, the network determinacy is given by the number of identifiable fluxes. The MATLAB code for calculability analysis is presented in Supplementary Material S4 – section II and III.

In this study, 18 input fluxes were obtained from extracellular measurements comprising cellular growth, uptake and secretion rates for glucose, lactate and amino acids. These fluxes are *tGln*, *tGlu*, *tGlc*, *tLac*, *tSer*, *tAla*, *tAsp*, *tAsn*, *muG6P*, *muGAP*, *muRu5P*, *muAcCoA*, *muSer*, *muAla*, *muAsp*, *muGln*, *muAsn*, and *muGlu*. Calculability analysis showed that only several intracellular fluxes that can be calculated with the extracellular free fluxes: *CIC*, *acl*, *asns*, *gdh*, *gs*, *ldh*, and *mAsn* (see section II for detailed information).

In accordance with the degree of freedom, additional 8 fluxes are required to solve the entire metabolic network. Apparently, the 8 fluxes are the intracellular fluxes. The calculability analysis showed that network determinacy (determined case) could be achieved by using a particular set of intracellular fluxes (*G6Pdh*, *phdgh*, *fGlyco*, *pepck*, *alt*, *me*, mem, *astm*) and the extracellular free fluxes (see section III for detailed information). These intracellular fluxes will be determined by fitting the ^{13}C enrichment profile of the respective metabolites involved in the biochemical reaction.

II. Identifiability Analysis case I

```
% Import metabolic network
S = xlsread('metabolic_model_CHO.xlsx');

flux_label = {'AGC1','CIC','DIC','GC1','MPC1','OGC','acl','adh','alt','altm',...
    'asnsm','ast','astm','csyn','pgi','pfk','fbpa','tpi','gapdh','eno','pkm',...
    'gdh','gs','idh','ldh','mAla','mAsn','mCO2','mdh','mdhc','me','mem','muAcCoA',...
    'muAla','muAsn','muAsp','muG6P','muGAP','muGln','muGlu','muP5P','muSer',...
    'pc','pdh','pepck','G6Pdh','rpi','tkt1','tkt2','phdgh','sds','tAla','tAsn',...
    'tAsp','tCO2','tGlc','tGln','tGlu','tGlyco','tLac','tSer'};

metabolite_label = {'Glum','Asp','Glu','Aspm','Cit','Malm','Citm','Mal','Pyrm',...
    'Pyr','aKG','aKGm','OAA','AcCoA','Asnm','OAAm','AcCoAm','F6P','G6P','FBP',...
    'DHAP','GAP','3PG','PEP','Gln','Lac','Alam','Ala','CO2','S7P','R5P','E4P',...
    'Ser','Asn','CO2m'};

% check identifiability
input_fluxes = [33 34 35 36 37 38 39 40 41 42 52 53 ...
    54 56 57 58 60 61];

unmeasured_fluxes = setdiff( 1:size(S,2), input_fluxes);
Sq = S(:,input_fluxes);
Su = S(:,unmeasured_fluxes);
RNS = null( Su );
k = find( all( [abs(RNS) zeros(size(RNS,1), 2)]' < 1e-6 ) );

% Print result
fprintf( 'Fluxes that can be determined from measurements: \n' )

for i=1:length( k )
    fprintf( '%s\n', flux_label{ unmeasured_fluxes(k(i))} );
end
```

```
Fluxes that can be determined from measurements:
CIC
acl
asnsm
gdh
gs
ldh
mAsn
```

Published with MATLAB® R2018b

III. Identifiability Analysis case II

```
% Import metabolic network
S = xlsread('metabolic_model_CHO.xlsx');

flux_label = {'AGC1','CIC','DIC','GC1','MPC1','OGC','acl','adh','alt','altm',...
    'asnsm','ast','astm','csyn','pgi','pfk','fbpa','tpi','gapdh','eno','pkm',...
    'gdh','gs','idh','ldh','mAla','mAsn','mCO2','mdh','mdhc','me','mem','muAcCoA',...
    'muAla','muAsn','muAsp','muG6P','muGAP','muGln','muGlu','muP5P','muSer',...
    'pc','pdh','pepck','G6Pdh','rpi','tkt1','tkt2','phdgh','sds','tAla','tAsn',...
    'tAsp','tCO2','tGlc','tGln','tGlu','tGlyco','tLac','tSer'};

metabolite_label = {'Glum','Asp','Glu','Aspm','Cit','Malm','Citm','Mal','Pyrm',...
    'Pyr','aKG','aKGm','OAA','AcCoA','Asnm','OAAm','AcCoAm','F6P','G6P','FBP',...
    'DHAP','GAP','3PG','PEP','Gln','Lac','Alam','Ala','CO2','S7P','R5P','E4P',...
    'Ser','Asn','CO2m'};

% check identifiability
input_fluxes = [9 13 31 33 34 35 36 37 38 39 40 41 42 43 45 46 50 52 53 ...
    54 56 57 58 59 60 61];

unmeasured_fluxes = setdiff( 1:size(S,2), input_fluxes);
Sq = S(:,input_fluxes);
Su = S(:,unmeasured_fluxes);
RNS = null( Su );
k = find( all( [abs(RNS) zeros(size(RNS,1), 2)]' < 1e-6 ) );

% Print result
fprintf( 'Fluxes that can be determined from measurements: \n' )

for i=1:length( k )
    fprintf( '%s\n', flux_label{ unmeasured_fluxes(k(i))} );
end
```

```
Fluxes that can be determined from measurements:
AGC1
CIC
DIC
GC1
MPC1
OGC
acl
adh
altm
asnsm
ast
csyn
pgi
pfk
fbpa
tpi
gapdh
eno
pkm
gdh
gs
idh
```

```
ldh
mAla
mAsn
mCO2
mdh
mdhc
mem
pdh
rpi
tkt1
tkt2
sds
tCO2
```

Published with MATLAB® R2018b

IV. *In silico* based 13C identifiability analysis (Experimental design)

To check whether the carbon tracer composition (75% [U-$^{13}C_6$]-D-glucose) allows sufficient identifiability of key fluxes (e.g. me_{cyt} identification using 13C Mal_{cyt} enrichment), *in silico* based studies were performed, investigating the impact of different of v_{me_cyt} and β_{me_cyt} (reversibility constant, as explained in the main text) to the Mal_{cyt} enrichment (Wiechert et al. 2015). By assuming real pool sizes measured by Junghans et al (2019) and by applying the planned labeling scenario of 75% [U-^{13}C]-D-glucose, realistic labeling patterns were created. Simulation results (cytosolic malate enrichment profile) are presented in Figure S4.1. As shown the obtained v_{me_cyt} (0.06 – 0.12 pmol cell^{-1} h^{-1}) and β_{me_cyt} (1 – 5) correspond to a very distinct Mal_{cyt} enrichment.

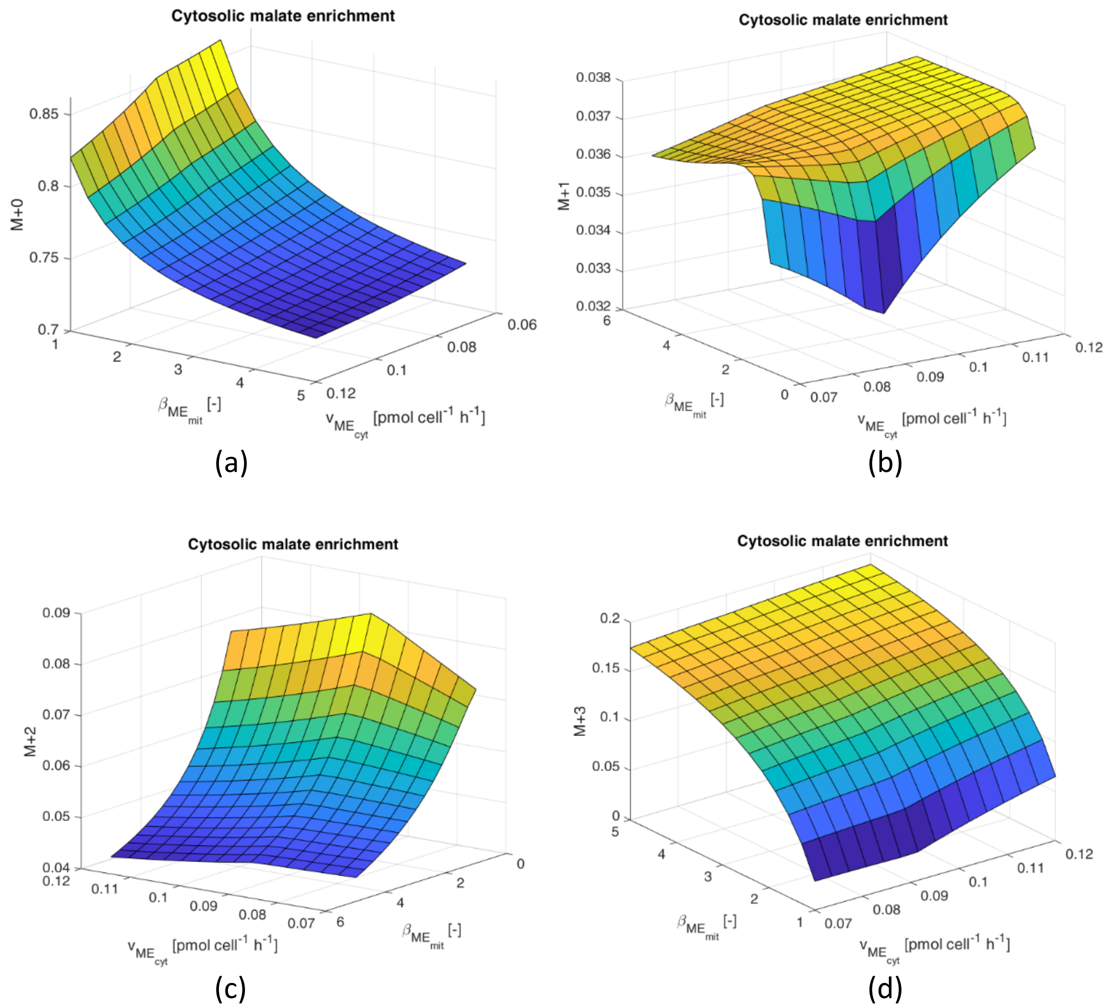

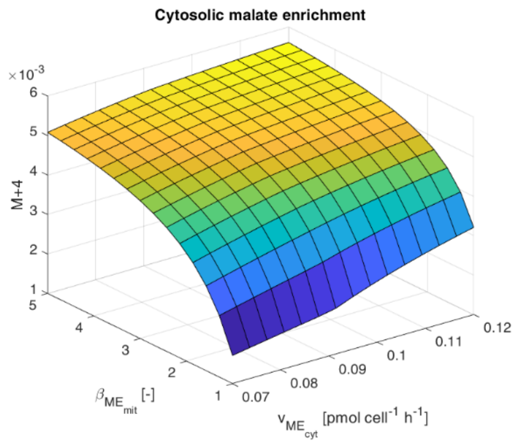

(e)

Figure S4.1. **Cytosolic malic enzyme identifiability analysis**: cytosolic malate (Mal_{cyt}) enrichment profile simulated using 75% [U-$^{13}C_6$]-D-glucose using various v_{me_cyt} (0.07 – 0.12 pmol cell^{-1} h^{-1})and β_{me_cyt} (1 – 5): (a) M+0, (b) M+1, (c) M+2, (d) M+3, (e) M+4 Mal_{cyt} mass-isotopomer species.

V. ^{13}C MFA identifiability analysis

To check whether ^{13}C labeling data (*in vivo*) may be used to identify targeted fluxes, ^{13}C simulations were performed *a priori*. Furthermore, input fluxes were assumed to be in realistic ranges, too. Figures S4.2 – S4.5 exemplify the good identifiability of intracellular fluxes when performing ^{13}C MFA. For the sake of brevity, only those fluxes are shown that were explicitly discussed in the manuscript. Accordingly, good identifiability is given for cytosolic and mitochondrial malic enzyme (Figure S4.2) and the shuttles AGC1, OGC, and DIC (Figure. S4.2 – S4.5).

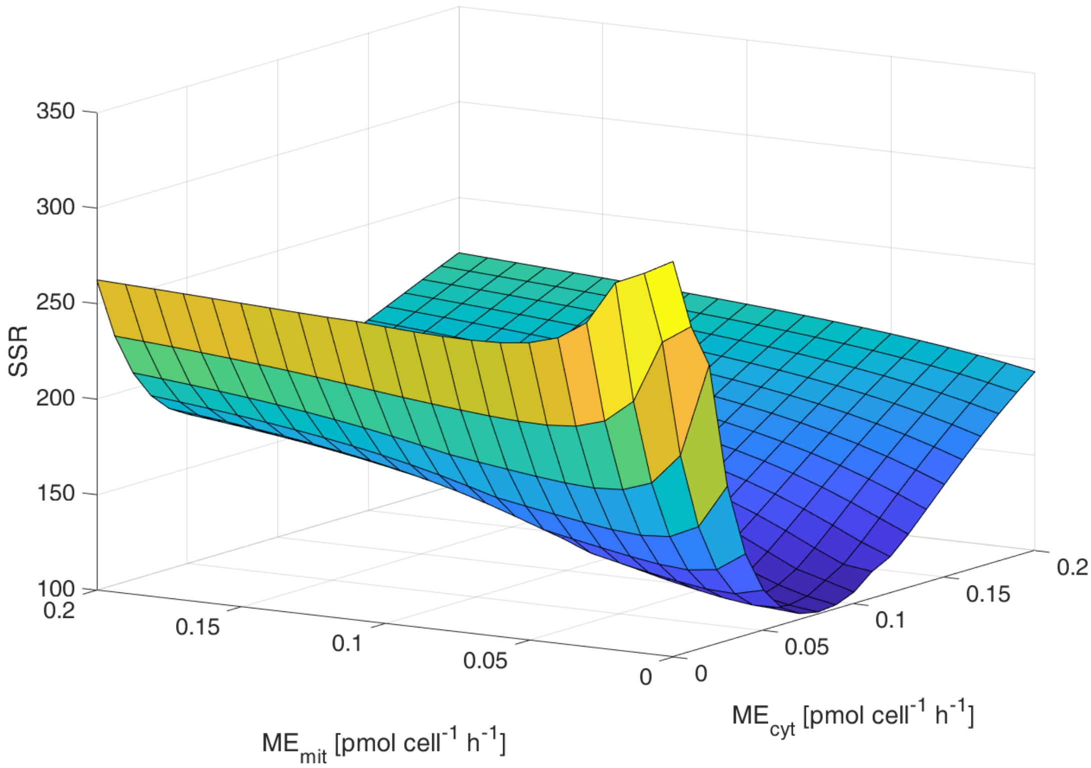

Figure S4.2. Identifiability analysis of malic enzymes. The figure indicates a global minimum for the cytosolic and mitochondrial malic enzymes activities reported in this study (v_{me_cyt} = 0.091 pmol cell^{-1} h^{-1} and v_{me_mit} = 0 pmol cell^{-1} h^{-1}).

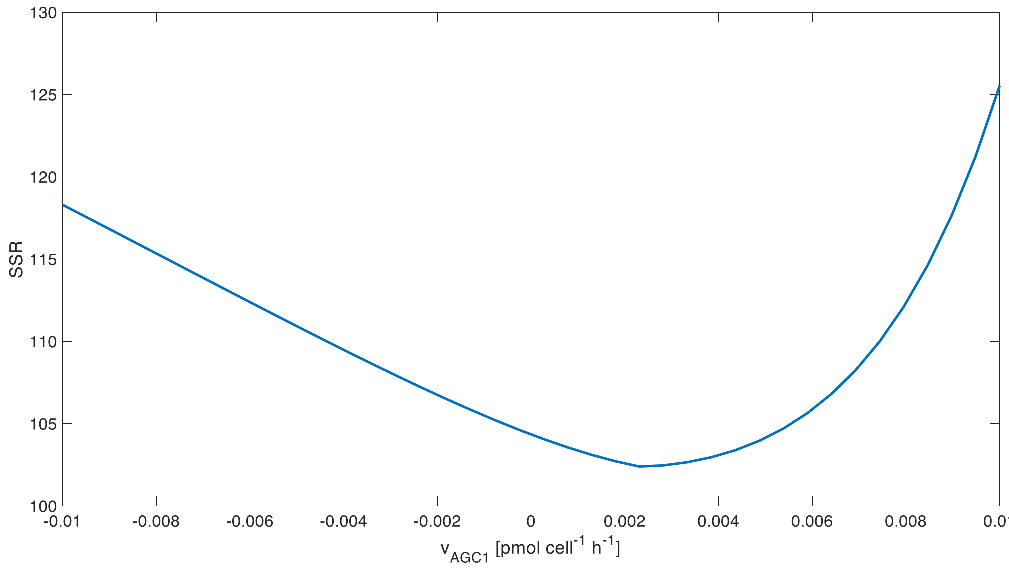

Figure S4.3. Identifiability analysis of aspartate-glutamate carrier (AGC1). The figure indicates a global minimum for the v_{AGC1} reported in this study (v_{AGC1} = 0.003 pmol cell^{-1} h^{-1}).

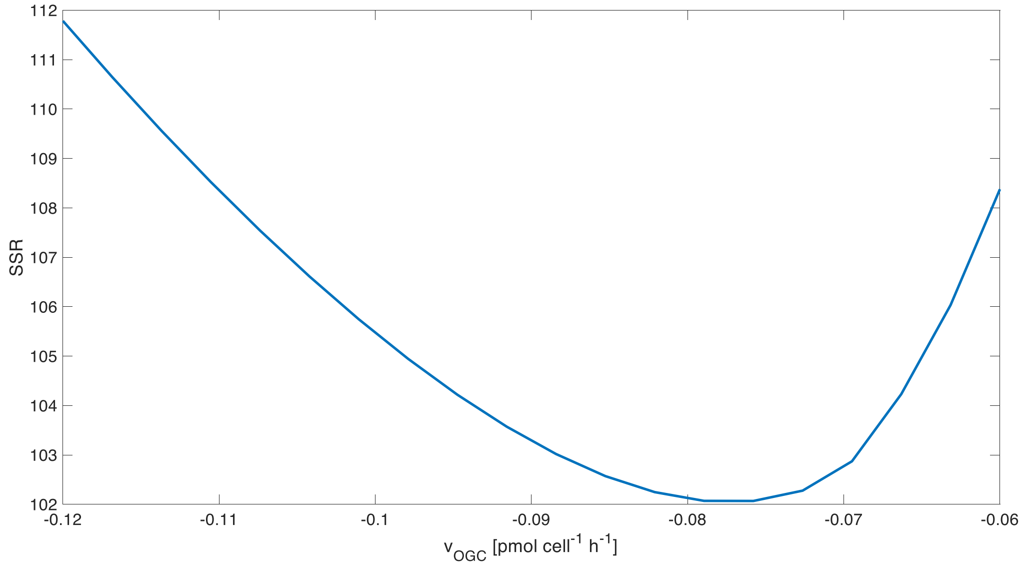

Figure S4.4. Identifiability analysis of oxo-glutarate-glutamate carrier (OGC). The figure indicates a global minimum for the v_{OGC} reported in this study (v_{OGC} = -0.0714 pmol cell^{-1} h^{-1}).

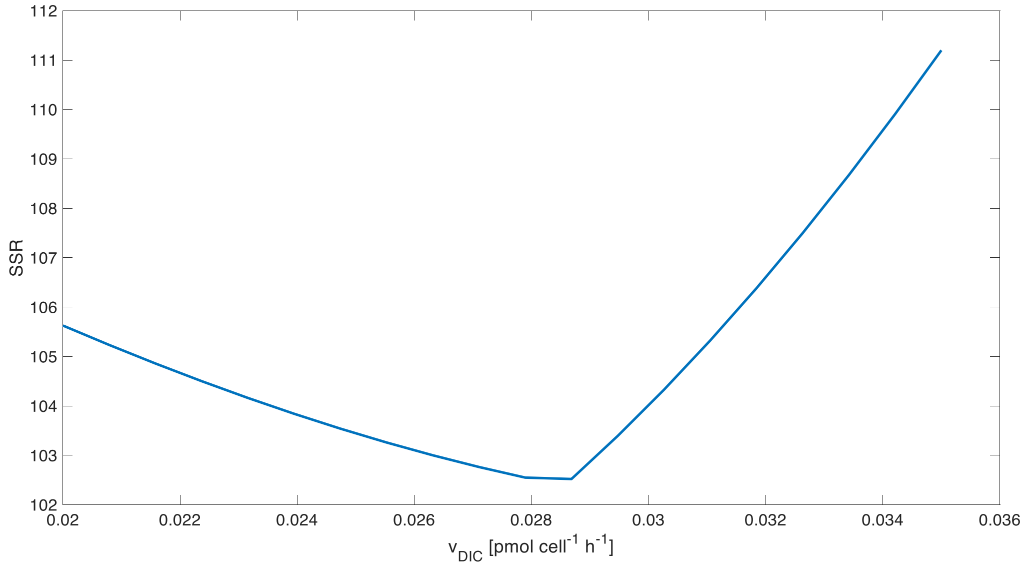

Figure S4.5. Identifiability analysis of malate carrier (DIC). The figure indicates a global minimum for the v_{DIC} reported in this study (v_{DIC} = 0.0287 pmol cell^{-1} h^{-1}).

Literature:

Wiechert, W., Niedenführ, S., & Nöh, K. (2015). A Primer to 13C metabolic flux analysis. *Fundamental Bioengineering*, 97-142.

METABOLIC NETWORK MODEL *S*

	AGC1	*CIC*	*DIC*	*GC1*	*MPC1*	*OGC*	*acl*	*adh*	*alt*	*altm*	*asnsm*	*ast*	*astm*	*csyn*	*pgi*	*pfk*	*fbpa*	*tpi*	*gapdh*	*eno*	*pkm*	*gdh*	*gs*	*idh*	*ldh*	*mAla*	*mAsn*	*mCO2*	*mdh*	*mdhc*	*me*	*mem*	*muAcCoA*	*muAla*
Glum	1	0	0	-1	0	0	0	0	0	1	0	0	1	0	0	0	0	0	0	0	0	-1	0	0	0	0	0	0	0	0	0	0	0	0
Asp	1	0	0	0	0	0	0	0	0	0	0	-1	0	0	0	0	0	0	0	0	0	0	0	0	0	0	0	0	0	0	0	0	0	0
Glu	-1	0	0	1	0	0	0	0	-1	0	0	1	0	0	0	0	0	0	0	0	0	0	1	0	0	0	0	0	0	0	0	0	0	0
Aspm	-1	0	0	0	0	0	0	0	0	0	1	0	-1	0	0	0	0	0	0	0	0	0	0	0	0	0	0	0	0	0	0	0	0	0
Cit	0	1	0	0	0	0	-1	0	0	0	0	0	0	0	0	0	0	0	0	0	0	0	0	0	0	0	0	0	0	0	0	0	0	0
Malm	0	1	-1	0	0	1	0	1	0	0	0	0	0	0	0	0	0	0	0	0	0	0	0	0	0	0	0	0	-1	0	0	-1	0	0
Citm	0	-1	0	0	0	0	0	0	0	0	0	0	0	1	0	0	0	0	0	0	0	0	0	-1	0	0	0	0	0	0	0	0	0	0
Mal	0	-1	1	0	0	-1	0	0	0	0	0	0	0	0	0	0	0	0	0	0	0	0	0	0	0	0	0	0	0	1	-1	0	0	0
Pyrm	0	0	0	0	1	0	0	0	0	1	0	0	0	0	0	0	0	0	0	0	0	0	0	0	0	0	0	0	0	0	0	1	0	0
Pyr	0	0	0	0	-1	0	0	0	-1	0	0	0	0	0	0	0	0	0	0	0	1	0	0	0	-1	0	0	0	0	0	1	0	0	0
aKG	0	0	0	0	0	1	0	0	1	0	0	-1	0	0	0	0	0	0	0	0	0	0	0	0	0	0	0	0	0	0	0	0	0	0
aKGm	0	0	0	0	0	-1	0	-1	0	-1	0	0	-1	0	0	0	0	0	0	0	0	1	0	1	0	0	0	0	0	0	0	0	0	0
OAA	0	0	0	0	0	0	1	0	0	0	0	1	0	0	0	0	0	0	0	0	0	0	0	0	0	0	0	0	0	-1	0	0	0	0
AcCoA	0	0	0	0	0	0	1	0	0	0	0	0	0	0	0	0	0	0	0	0	0	0	0	0	0	0	0	0	0	0	0	0	-1	0
Asnm	0	0	0	0	0	0	0	0	0	0	-1	0	0	0	0	0	0	0	0	0	0	0	0	0	0	0	1	0	0	0	0	0	0	0
OAAm	0	0	0	0	0	0	0	0	0	0	0	0	1	-1	0	0	0	0	0	0	0	0	0	0	0	0	0	0	1	0	0	0	0	0
AcCoAm	0	0	0	0	0	0	0	0	0	0	0	0	0	-1	0	0	0	0	0	0	0	0	0	0	0	0	0	0	0	0	0	0	0	0
F6P	0	0	0	0	0	0	0	0	0	0	0	0	0	0	1	-1	0	0	0	0	0	0	0	0	0	0	0	0	0	0	0	0	0	0
G6P	0	0	0	0	0	0	0	0	0	0	0	0	0	0	-1	0	0	0	0	0	0	0	0	0	0	0	0	0	0	0	0	0	0	0
FBP	0	0	0	0	0	0	0	0	0	0	0	0	0	0	0	1	-1	0	0	0	0	0	0	0	0	0	0	0	0	0	0	0	0	0
DHAP	0	0	0	0	0	0	0	0	0	0	0	0	0	0	0	0	1	-1	0	0	0	0	0	0	0	0	0	0	0	0	0	0	0	0
GAP	0	0	0	0	0	0	0	0	0	0	0	0	0	0	0	0	1	1	-1	0	0	0	0	0	0	0	0	0	0	0	0	0	0	0
3PG	0	0	0	0	0	0	0	0	0	0	0	0	0	0	0	0	0	0	1	-1	0	0	0	0	0	0	0	0	0	0	0	0	0	0
PEP	0	0	0	0	0	0	0	0	0	0	0	0	0	0	0	0	0	0	0	1	-1	0	0	0	0	0	0	0	0	0	0	0	0	0
Gln	0	0	0	0	0	0	0	0	0	0	0	0	0	0	0	0	0	0	0	0	0	0	-1	0	0	0	0	0	0	0	0	0	0	0
Lac	0	0	0	0	0	0	0	0	0	0	0	0	0	0	0	0	0	0	0	0	0	0	0	0	1	0	0	0	0	0	0	0	0	0
Alam	0	0	0	0	0	0	0	0	0	-1	0	0	0	0	0	0	0	0	0	0	0	0	0	0	0	1	0	0	0	0	0	0	0	0
Ala	0	0	0	0	0	0	0	0	1	0	0	0	0	0	0	0	0	0	0	0	0	0	0	0	0	-1	0	0	0	0	0	0	0	-1
CO2	0	0	0	0	0	0	0	0	0	0	0	0	0	0	0	0	0	0	0	0	0	0	0	0	0	0	0	1	0	0	1	0	0	0
S7P	0	0	0	0	0	0	0	0	0	0	0	0	0	0	0	0	0	0	0	0	0	0	0	0	0	0	0	0	0	0	0	0	0	0
R5P	0	0	0	0	0	0	0	0	0	0	0	0	0	0	0	0	0	0	0	0	0	0	0	0	0	0	0	0	0	0	0	0	0	0
E4P	0	0	0	0	0	0	0	0	0	0	0	0	0	0	0	0	0	0	0	0	0	0	0	0	0	0	0	0	0	0	0	0	0	0
Ser	0	0	0	0	0	0	0	0	0	0	0	0	0	0	0	0	0	0	0	0	0	0	0	0	0	0	0	0	0	0	0	0	0	0
Asn	0	0	0	0	0	0	0	0	0	0	0	0	0	0	0	0	0	0	0	0	0	0	0	0	0	0	-1	0	0	0	0	0	0	0
CO2m	0	0	0	0	0	0	0	1	0	0	0	0	0	0	0	0	0	0	0	0	0	0	0	1	0	0	0	-1	0	0	0	1	0	0

METABOLIC NETWORK MODEL *S* (continue)

cont…	*muAsn*	*muAsp*	*muG6P*	*muGAP*	*muGln*	*muGlu*	*muP5P*	*muSer*	*pc*	*pdh*	*pepck*	*G6Pdh*	*rpi*	*tkt1*	*tkt2*	*phdgh*	*sds*	*tAla*	*tAsn*	*tAsp*	*tCO2*	*tGlc*	*tGln*	*tGlu*	*tGlyco*	*tLac*	*tSer*
Glum	0	0	0	0	0	0	0	0	0	0	0	0	0	0	0	0	0	0	0	0	0	0	0	0	0	0	0
Asp	0	-1	0	0	0	0	0	0	0	0	0	0	0	0	0	0	0	0	0	-1	0	0	0	0	0	0	0
Glu	0	0	0	0	0	-1	0	0	0	0	0	0	0	0	0	0	0	0	0	0	0	0	0	-1	0	0	0
Aspm	0	0	0	0	0	0	0	0	0	0	0	0	0	0	0	0	0	0	0	0	0	0	0	0	0	0	0
Cit	0	0	0	0	0	0	0	0	0	0	0	0	0	0	0	0	0	0	0	0	0	0	0	0	0	0	0
Malm	0	0	0	0	0	0	0	0	0	0	0	0	0	0	0	0	0	0	0	0	0	0	0	0	0	0	0
Citm	0	0	0	0	0	0	0	0	0	0	0	0	0	0	0	0	0	0	0	0	0	0	0	0	0	0	0
Mal	0	0	0	0	0	0	0	0	0	0	0	0	0	0	0	0	0	0	0	0	0	0	0	0	0	0	0
Pyrm	0	0	0	0	0	0	0	0	-1	-1	0	0	0	0	0	0	0	0	0	0	0	0	0	0	0	0	0
Pyr	0	0	0	0	0	0	0	0	0	0	0	0	0	0	0	0	1	0	0	0	0	0	0	0	0	0	0
aKG	0	0	0	0	0	0	0	0	0	0	0	0	0	0	0	0	0	0	0	0	0	0	0	0	0	0	0
aKGm	0	0	0	0	0	0	0	0	0	0	0	0	0	0	0	0	0	0	0	0	0	0	0	0	0	0	0
OAA	0	0	0	0	0	0	0	0	0	0	-1	0	0	0	0	0	0	0	0	0	0	0	0	0	0	0	0
AcCoA	0	0	0	0	0	0	0	0	0	0	0	0	0	0	0	0	0	0	0	0	0	0	0	0	0	0	0
Asnm	0	0	0	0	0	0	0	0	0	0	0	0	0	0	0	0	0	0	0	0	0	0	0	0	0	0	0
OAAm	0	0	0	0	0	0	0	0	1	0	0	0	0	0	0	0	0	0	0	0	0	0	0	0	0	0	0
AcCoAm	0	0	0	0	0	0	0	0	0	1	0	0	0	0	0	0	0	0	0	0	0	0	0	0	0	0	0
F6P	0	0	0	0	0	0	0	0	0	0	0	0	0	1	1	0	0	0	0	0	0	0	0	0	0	0	0
G6P	0	0	-1	0	0	0	0	0	0	0	0	-1	0	0	0	0	0	0	0	0	0	1	0	0	1	0	0
FBP	0	0	0	0	0	0	0	0	0	0	0	0	0	0	0	0	0	0	0	0	0	0	0	0	0	0	0
DHAP	0	0	0	0	0	0	0	0	0	0	0	0	0	0	0	0	0	0	0	0	0	0	0	0	0	0	0
GAP	0	0	0	-1	0	0	0	0	0	0	0	0	1	1	-1	0	0	0	0	0	0	0	0	0	0	0	0
3PG	0	0	0	0	0	0	0	0	0	0	0	0	0	0	0	-1	0	0	0	0	0	0	0	0	0	0	0
PEP	0	0	0	0	0	0	0	0	0	0	1	0	0	0	0	0	0	0	0	0	0	0	0	0	0	0	0
Gln	0	0	0	0	-1	0	0	0	0	0	0	0	0	0	0	0	0	0	0	0	0	0	1	0	0	0	0
Lac	0	0	0	0	0	0	0	0	0	0	0	0	0	0	0	0	0	0	0	0	0	0	0	0	0	-1	0
Alam	0	0	0	0	0	0	0	0	0	0	0	0	0	0	0	0	0	0	0	0	0	0	0	0	0	0	0
Ala	0	0	0	0	0	0	0	0	0	0	0	0	0	0	0	0	0	-1	0	0	0	0	0	0	0	0	0
CO2	0	0	0	0	0	0	0	0	0	0	1	1	0	0	0	0	0	0	0	0	-1	0	0	0	0	0	0
S7P	0	0	0	0	0	0	0	0	0	0	0	0	1	0	-1	0	0	0	0	0	0	0	0	0	0	0	0
R5P	0	0	0	0	0	0	-1	0	0	0	0	1	-2	-1	0	0	0	0	0	0	0	0	0	0	0	0	0
E4P	0	0	0	0	0	0	0	0	0	0	0	0	0	-1	1	0	0	0	0	0	0	0	0	0	0	0	0
Ser	0	0	0	0	0	0	0	-1	0	0	0	0	0	0	0	1	-1	0	0	0	0	0	0	0	0	0	1
Asn	-1	0	0	0	0	0	0	0	0	0	0	0	0	0	0	0	0	0	1	0	0	0	0	0	0	0	0
CO2m	0	0	0	0	0	0	0	0	-1	1	0	0	0	0	0	0	0	0	0	0	0	0	0	0	0	0	0
