# Supplementary material for: Compartment-specific metabolome labeling enables the identification of subcellular fluxes that may serve as promising metabolic engineering targets in CHO cells
Source: Bioprocess Biosyst Eng. 2021 Sep 30;44(12):2567–78. doi: 10.1007/s00449-021-02628-1 (PMC8536584; doi:10.1007/s00449-021-02628-1)
Supplement: Supplementary file 5 — Supplementary file5 (PDF 73 kb) [file 449_2021_2628_MOESM5_ESM.pdf]

COMPARTMENT-SPECIFIC 13C MFA

	m+0			m+1			m+2			m+3			m+4			m+5			m+6		
MID	meas	meas_std	sim	meas	meas_std	sim	meas	meas_std	sim	meas	meas_std	sim	meas	meas_std	sim	meas	meas_std	sim	meas	meas_std	sim
G6P_T0	0.986	0.025	0.986	0.000	0.025	0.000	0.021	0.025	0.021	0.000	0.025	0.000	0.000	0.025	0.000	0.000	0.025	0.000	0.000	0.025	0.000
G6P_T1	0.278	0.025	0.318	0.013	0.025	0.002	0.029	0.025	0.010	0.058	0.025	0.084	0.033	0.025	0.004	0.051	0.025	0.004	0.539	0.025	0.585
G6P_T2	0.278	0.025	0.310	0.017	0.025	0.002	0.033	0.025	0.009	0.063	0.025	0.080	0.039	0.025	0.005	0.056	0.025	0.005	0.516	0.025	0.596
P5P_T0	0.976	0.010	0.976	0.006	0.010	0.006	0.019	0.010	0.019	0.020	0.020	0.000	0.000	0.020	0.000	0.000	0.020	0.000			
P5P_T1	0.265	0.026	0.260	0.062	0.010	0.040	0.095	0.010	0.110	0.110	0.014	0.134	0.000	0.020	0.013	0.470	0.029	0.443			
P5P_T2	0.248	0.029	0.228	0.062	0.010	0.043	0.094	0.020	0.116	0.110	0.010	0.121	0.000	0.020	0.020	0.488	0.021	0.473			
PEP_T0	0.958	0.010	0.958	0.011	0.010	0.011	0.032	0.010	0.032	0.000	0.010	0.000									
PEP_T1	0.270	0.030	0.366	0.029	0.010	0.004	0.076	0.016	0.009	0.625	0.021	0.621									
PEP_T2	0.256	0.029	0.352	0.031	0.010	0.005	0.084	0.021	0.009	0.630	0.017	0.634									
Pyr_T0	0.932	0.020	0.932	0.061	0.025	0.061	0.008	0.010	0.008	0.000	0.010	0.000									
Pyr_T1	0.748	0.020	0.780	0.056	0.025	0.007	0.022	0.010	0.009	0.175	0.015	0.204									
Pyr_T2	0.665	0.025	0.727	0.056	0.025	0.010	0.027	0.010	0.013	0.252	0.019	0.250									
Pyr_m_T0	0.933	0.025	0.933	0.059	0.025	0.059	0.008	0.010	0.008	0.000	0.010	0.000									
Pyr_m_T1	0.781	0.025	0.824	0.062	0.025	0.005	0.020	0.010	0.007	0.138	0.017	0.163									
Pyr_m_T2	0.711	0.025	0.774	0.057	0.025	0.009	0.026	0.010	0.011	0.207	0.021	0.207									
Ser_T0	0.971	0.011	0.971	0.008	0.010	0.008	0.007	0.010	0.007	0.014	0.010	0.014									
Ser_T1	0.959	0.025	0.957	0.008	0.011	0.000	0.007	0.041	0.001	0.027	0.049	0.042									
Ser_T2	0.939	0.014	0.956	0.010	0.010	0.000	0.010	0.010	0.001	0.041	0.010	0.043									
Ala_T0	1.000	0.010	1.000	0.000	0.010	0.000	0.000	0.010	0.000	0.000	0.001	0.000									
Ala_T1	0.957	0.010	0.955	0.004	0.010	0.001	0.002	0.010	0.002	0.038	0.010	0.043									
Ala_T2	0.905	0.019	0.914	0.005	0.010	0.003	0.009	0.010	0.004	0.081	0.014	0.079									
Ala_m_T0	1.000	0.010	1.000	0.000	0.010	0.000	0.000	0.010	0.000	0.000	0.010	0.000									
Ala_m_T1	0.975	0.010	0.965	0.000	0.010	0.001	0.000	0.010	0.001	0.035	0.010	0.033									
Ala_m_T2	0.902	0.030	0.925	0.010	0.010	0.002	0.016	0.010	0.003	0.088	0.020	0.070									
aKG_m_T0	0.933	0.025	0.933	0.065	0.025	0.065	0.002	0.025	0.002	0.000	0.025	0.000	0.000	0.025	0.000	0.000	0.025	0.000			
aKG_m_T1	0.873	0.025	0.883	0.066	0.025	0.024	0.063	0.025	0.082	0.000	0.025	0.009	0.000	0.025	0.001	0.000	0.025	0.000			
aKG_m_T2	0.822	0.025	0.840	0.084	0.025	0.039	0.092	0.025	0.104	0.003	0.025	0.014	0.000	0.025	0.003	0.000	0.025	0.000			
aKG_T0	0.933	0.025	0.933	0.059	0.025	0.059	0.008	0.025	0.008	0.000	0.025	0.000	0.000	0.025	0.000	0.000	0.025	0.000			
aKG_T1	0.864	0.025	0.894	0.069	0.025	0.022	0.060	0.025	0.074	0.007	0.025	0.008	0.000	0.025	0.001	0.000	0.025	0.000			
aKG_T2	0.818	0.025	0.856	0.075	0.025	0.035	0.091	0.025	0.094	0.017	0.025	0.013	0.000	0.025	0.003	0.000	0.025	0.000			
Mal_m_T0	0.993	0.025	0.993	0.000	0.025	0.000	0.010	0.025	0.010	0.000	0.025	0.000	0.000	0.025	0.000						
Mal_m_T1	0.924	0.025	0.893	0.014	0.025	0.021	0.057	0.025	0.067	0.004	0.025	0.021	0.001	0.025	0.001						
Mal_m_T2	0.866	0.028	0.852	0.026	0.025	0.033	0.098	0.025	0.087	0.008	0.025	0.028	0.002	0.025	0.002						
Mal_T0	0.993	0.010	0.993	0.000	0.010	0.000	0.009	0.010	0.009	0.000	0.020	0.000	0.000	0.020	0.000						
Mal_T1	0.934	0.020	0.894	0.014	0.010	0.020	0.048	0.010	0.065	0.004	0.020	0.022	0.000	0.020	0.001						
Mal_T2	0.879	0.020	0.852	0.027	0.010	0.033	0.084	0.010	0.085	0.009	0.020	0.030	0.001	0.020	0.002						
Asp_T0	0.987	0.010	0.987	0.006	0.010	0.006	0.007	0.010	0.007	0.000	0.010	0.000	0.000	0.010	0.000						
Asp_T1	0.945	0.010	0.939	0.016	0.010	0.011	0.035	0.010	0.029	0.003	0.010	0.021	0.000	0.010	0.000						
Asp_T2	0.911	0.011	0.906	0.025	0.010	0.023	0.056	0.010	0.042	0.007	0.010	0.029	0.001	0.010	0.001						
Asp_m_T0	0.990	0.010	0.990	0.005	0.010	0.005	0.006	0.020	0.006	0.000	0.010	0.000	0.000	0.010	0.000						
Asp_m_T1	0.962	0.010	0.969	0.015	0.010	0.003	0.022	0.020	0.005	0.001	0.015	0.023	0.000	0.010	0.000						
Asp_m_T2	0.960	0.010	0.958	0.015	0.010	0.005	0.023	0.020	0.007	0.002	0.015	0.029	0.000	0.010	0.001						

NON-COMPARTMENTED 13C MFA

MID	m+0			m+1			m+2			m+3			m+4			m+5			m+6		
	meas	meas_std	sim	meas	meas_std	sim	meas	meas_std	sim	meas	meas_std	sim	meas	meas_std	sim	meas	meas_std	sim	meas	meas_std	sim
G6P_T0	0.986	0.025	0.986	0.000	0.025	0.000	0.021	0.025	0.021	0.000	0.025	0.000	0.000	0.025	0.000	0.000	0.025	0.000	0.000	0.025	0.000
G6P_T1	0.278	0.025	0.318	0.013	0.025	0.002	0.029	0.025	0.010	0.058	0.025	0.084	0.033	0.025	0.004	0.051	0.025	0.004	0.539	0.025	0.585
G6P_T2	0.278	0.025	0.310	0.017	0.025	0.002	0.033	0.025	0.009	0.063	0.025	0.080	0.039	0.025	0.005	0.056	0.025	0.005	0.516	0.025	0.596
P5P_T0	0.976	0.010	0.976	0.006	0.010	0.006	0.019	0.010	0.019	0.020	0.020	0.000	0.000	0.020	0.000	0.000	0.020	0.000			
P5P_T1	0.265	0.026	0.260	0.062	0.010	0.040	0.095	0.010	0.110	0.110	0.014	0.134	0.000	0.020	0.013	0.470	0.029	0.443			
P5P_T2	0.248	0.029	0.228	0.062	0.010	0.043	0.094	0.020	0.116	0.110	0.010	0.121	0.000	0.020	0.020	0.488	0.021	0.473			
PEP_T0	0.958	0.025	0.958	0.011	0.025	0.011	0.032	0.025	0.032	0.000	0.025	0.000									
PEP_T1	0.270	0.300	0.366	0.029	0.025	0.004	0.076	0.300	0.009	0.625	0.025	0.621									
PEP_T2	0.256	0.300	0.352	0.031	0.025	0.005	0.084	0.300	0.009	0.630	0.025	0.634									
Pyr_T0	0.932	0.020	0.932	0.061	0.025	0.061	0.008	0.010	0.008	0.000	0.010	0.000									
Pyr_T1	0.748	0.020	0.784	0.056	0.025	0.010	0.022	0.010	0.009	0.175	0.015	0.196									
Pyr_T2	0.665	0.025	0.731	0.056	0.025	0.013	0.027	0.010	0.012	0.252	0.019	0.243									
Ser_T0	0.971	0.011	0.971	0.008	0.010	0.008	0.007	0.010	0.007	0.014	0.010	0.014									
Ser_T1	0.959	0.025	0.957	0.008	0.011	0.000	0.007	0.041	0.001	0.027	0.049	0.042									
Ser_T2	0.939	0.014	0.956	0.010	0.010	0.000	0.010	0.010	0.001	0.041	0.010	0.043									
Ala_T0	1.000	0.025	1.000	0.000	0.025	0.000	0.000	0.025	0.000	0.000	0.025	0.000									
Ala_T1	0.957	0.025	0.864	0.004	0.025	0.035	0.002	0.025	0.027	0.038	0.025	0.073									
Ala_T2	0.905	0.025	0.854	0.005	0.025	0.039	0.009	0.025	0.029	0.081	0.025	0.078									
aKG_T0	0.933	0.025	0.933	0.059	0.025	0.064	0.008	0.025	0.002	0.000	0.025	0.000	0.000	0.025	0.000	0.000	0.025	0.000			
aKG_T1	0.864	0.025	0.874	0.069	0.025	0.042	0.060	0.025	0.069	0.007	0.025	0.013	0.000	0.025	0.002	0.000	0.025	0.000			
aKG_T2	0.818	0.025	0.841	0.075	0.025	0.058	0.091	0.025	0.079	0.017	0.025	0.017	0.000	0.025	0.004	0.000	0.025	0.000			
Mal_T0	0.993	0.010	0.993	0.000	0.010	0.000	0.009	0.010	0.010	0.000	0.020	0.000	0.000	0.020	0.000						
Mal_T1	0.934	0.020	0.885	0.014	0.010	0.030	0.048	0.010	0.067	0.004	0.020	0.019	0.000	0.020	0.002						
Mal_T2	0.879	0.020	0.863	0.027	0.010	0.036	0.084	0.010	0.078	0.009	0.020	0.023	0.001	0.020	0.003						
Asp_T0	0.987	0.010	0.988	0.006	0.010	0.005	0.007	0.010	0.007	0.000	0.010	0.000	0.000	0.010	0.000						
Asp_T1	0.945	0.010	0.938	0.016	0.010	0.020	0.035	0.010	0.029	0.003	0.010	0.013	0.000	0.010	0.000						
Asp_T2	0.911	0.011	0.884	0.025	0.010	0.044	0.056	0.010	0.049	0.007	0.010	0.023	0.001	0.010	0.001						
