# Supplementary material for: Compartment-specific metabolome labeling enables the identification of subcellular fluxes that may serve as promising metabolic engineering targets in CHO cells
Source: Bioprocess Biosyst Eng. 2021 Sep 30;44(12):2567–78. doi: 10.1007/s00449-021-02628-1 (PMC8536584; doi:10.1007/s00449-021-02628-1)
Supplement: Supplementary file 6 — Supplementary file6 (DOCX 178 kb) [file 449_2021_2628_MOESM6_ESM.docx]

Supplementary Material S6

The same cell line (CHO-DP12), cultivation condition (batch using TC42 media), and analytical tools were used to reproduce the dataset of Junghans et al. (2019). The additional dataset was used to perform a similar study as in the main text. The results are presented in Figure S6-1 to S6-3.


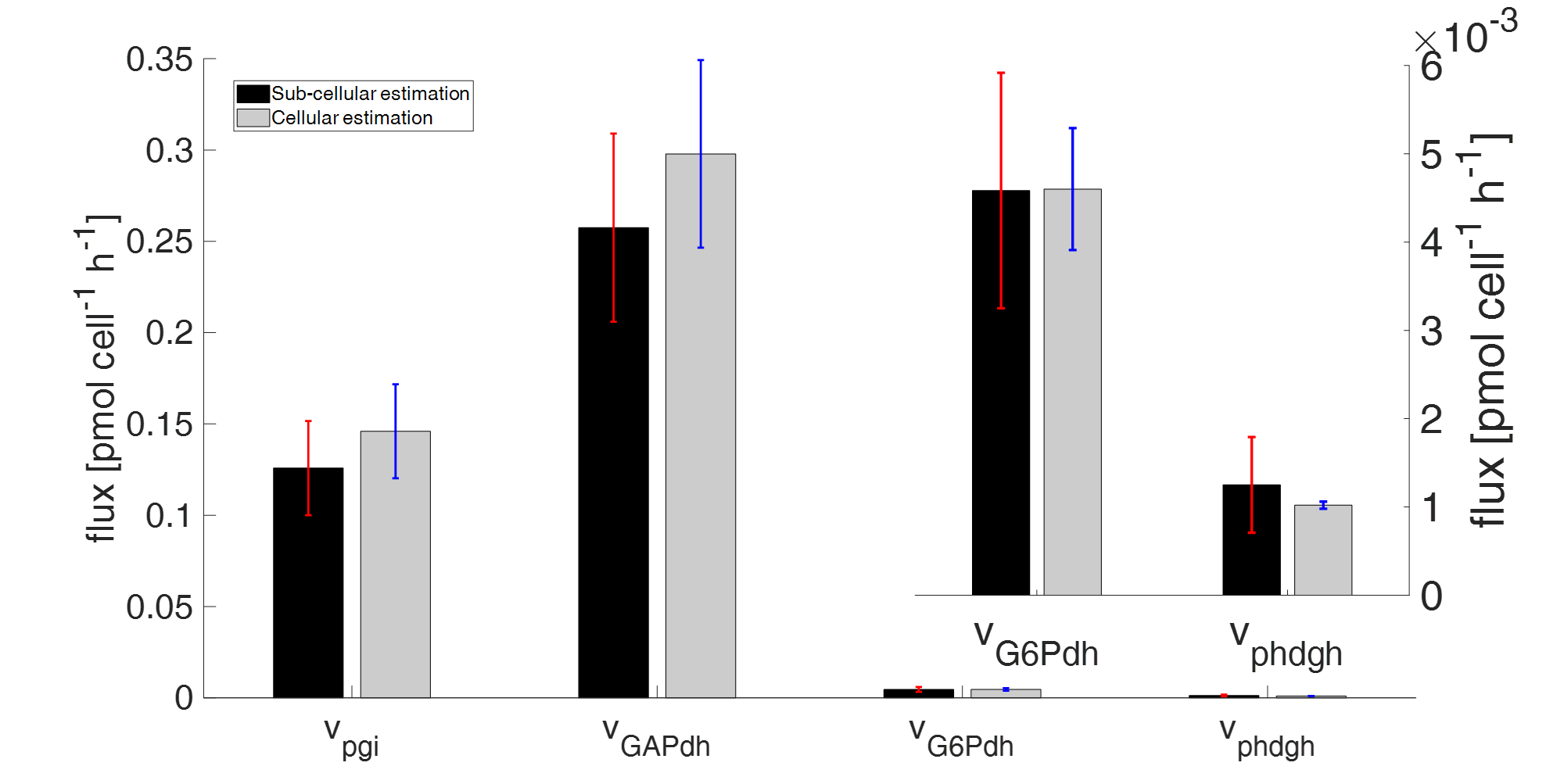


Figure S6-1: Fluxes of biochemical reactions involving single-compartment metabolites (* indicates significance p < 0.05)


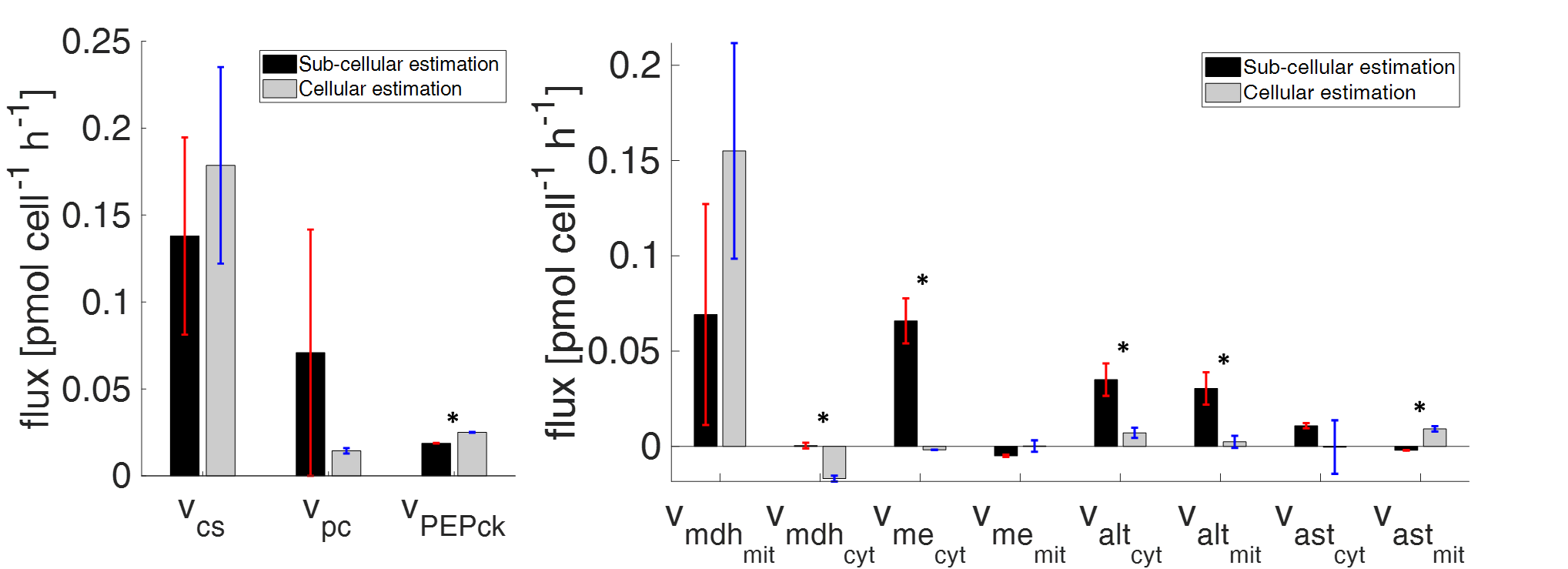


Figure S6-1: Fluxes of biochemical reactions involving multi-compartment metabolites (* indicates significance p < 0.05)


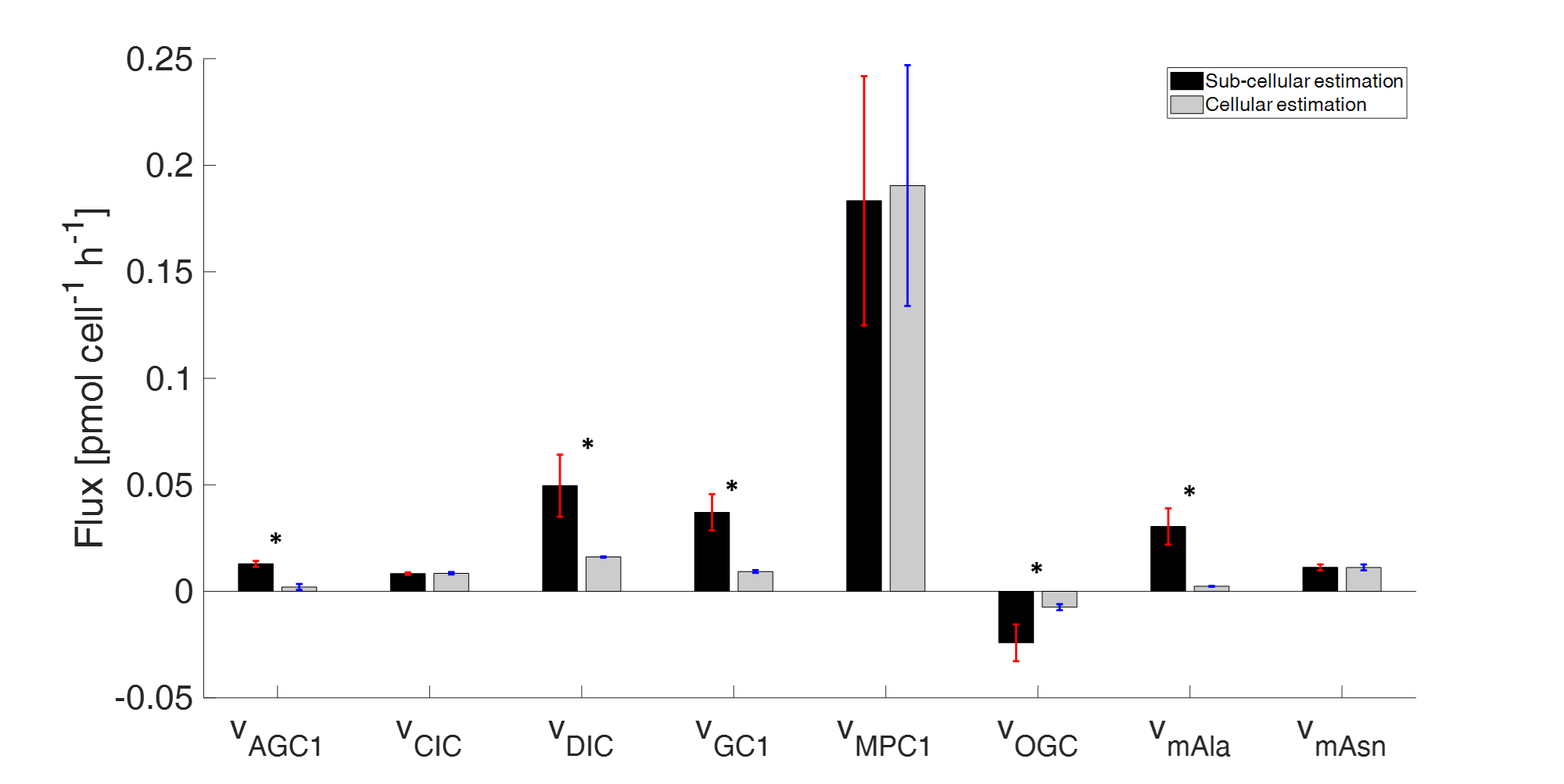


Figure S6-1: Mitochondrial carrier fluxes (* indicates significance p < 0.05)
